# Role of Integrins in Resistance to Therapies Targeting Growth Factor Receptors in Cancer

**DOI:** 10.3390/cancers11050692

**Published:** 2019-05-17

**Authors:** Elisabete Cruz da Silva, Monique Dontenwill, Laurence Choulier, Maxime Lehmann

**Affiliations:** UMR 7021 CNRS, Laboratoire de Bioimagerie et Pathologies, Tumoral Signaling and Therapeutic Targets, Université de Strasbourg, Faculté de Pharmacie, 67401 Illkirch, France; elisabete.cruz-da-silva@etu.unistra.fr (E.C.d.S.); monique.dontenwill@unistra.fr (M.D.); laurence.choulier@unistra.fr (L.C.)

**Keywords:** integrin, focal adhesion kinase, therapy resistance, tyrosine kinase inhibitors, cancer-associated fibroblasts, mechanotransduction, EGFR, c-MET

## Abstract

Integrins contribute to cancer progression and aggressiveness by activating intracellular signal transduction pathways and transducing mechanical tension forces. Remarkably, these adhesion receptors share common signaling networks with receptor tyrosine kinases (RTKs) and support their oncogenic activity, thereby promoting cancer cell proliferation, survival and invasion. During the last decade, preclinical studies have revealed that integrins play an important role in resistance to therapies targeting RTKs and their downstream pathways. A remarkable feature of integrins is their wide-ranging interconnection with RTKs, which helps cancer cells to adapt and better survive therapeutic treatments. In this context, we should consider not only the integrins expressed in cancer cells but also those expressed in stromal cells, since these can mechanically increase the rigidity of the tumor microenvironment and confer resistance to treatment. This review presents some of these mechanisms and outlines new treatment options for improving the efficacy of therapies targeting RTK signaling.

## 1. Introduction

Many tumors initially respond to targeted therapies before resistance appears. The mechanisms that sustain tumor cells between initial response and disease progression are not well understood. Understanding drug resistance is urgently needed in cancer therapy. The interaction between cancer cells and the microenvironment (the extracellular matrix (ECM), fibroblasts, endothelial cells, and immune cells) is essential to cell survival, proliferation and migration [1,2]. Be it through physiological mechanisms or remodeling after therapy, the tumor microenvironment provides a safe haven that promotes the emergence of resistance.

The ECM alone can induce tumor cell resistance to treatment [3]. Integrins, a family of cell surface receptors, play an important role in the interaction with the ECM. The integrin family comprises 24 different receptors made up of heterodimers of 18 alpha (α) and 8 beta (β) subunits, each of which binds to one or more ECM ligands. Integrins are involved in cellular adhesion to the ECM and in intercellular cohesion. Integrin biochemical and mechanical signaling regulates cell survival, proliferation, differentiation, migration, adhesion, apoptosis, anoikis, polarity and stemness [4,5,6]. Since integrins do not have enzymatic activity, once they bind to a ligand, they recruit cytoplasmic kinases such as focal adhesion kinases (FAKs). These, once recruited, autophosphorylate and present a docking site for the proto-oncogene tyrosine-protein kinase Src [7]. The FAK/Src complex activates the NF–kB (nuclear factor–kappa B), MAPK (mitogen-activated protein kinase) and PI3K (phosphoinositide 3-kinases) pathways. These signaling pathways are redundant with the receptor tyrosine kinase (RTK) signaling pathways. RTKs are families of surface receptors with tyrosine kinase activity that bind to growth factors, cytokines and hormones. RTK signaling pathways regulate cell growth, differentiation, metabolism and apoptosis in response to growth factor stimulation of cross-activation by co-receptors such as integrins. In normal cells, RTK function is tightly regulated. However, in cancer, mutations, overexpression, autocrine/paracrine stimulation and aberrant degradation lead to RTK constitutive activation and consequently to tumor formation and progression [8,9].

Integrins cooperate with several RTKs, such as epidermal growth factor receptor (EGFR), c-Met, platelet-derived growth factor receptor (PDGFR), insulin-like growth factor receptor (IGFR) and vascular endothelial growth factor receptor (VEGFR). This cooperation promotes solid tumor progression and aggressiveness as well as contributing to therapy resistance, be it to chemotherapy, radiotherapy or targeted therapy. Integrin/RTK crosstalk has been well described in several reviews [4,5]. In recent decades, integrins have emerged as new players in resistance to RTK-targeted therapies. The purpose of this review is to present a synthesis of the literature and to explore the diversity of the mechanisms by which integrins are able to counteract RTK-targeted therapies (Table 1). New promising therapeutic approaches resulting from these discoveries will be also discussed.

## 2. β1 Integrins

β1 integrins form heterodimers with no less than 12 of the 18 known alpha subunits, and thus represent the largest integrin subgroup. β1 integrins are overexpressed in solid tumors such as breast carcinoma, lung carcinoma and head and neck squamous cell carcinoma (HNSCC) [32,33,34]. In cancer cells, β1 integrins are associated with proliferative signaling, trigger cell death resistance, induce angiogenesis and activate cell migration and the metastatic cascade [35,36,37,38,39]. β1 integrins contribute to chemotherapy resistance [38,40,41,42,43,44,45,46,47,48,49,50] and promote radiotherapy resistance in HNSCC [51,52,53,54], breast carcinoma [55,56], laryngeal carcinoma [57,58], and glioma [59,60]. Based on these observations, β1 integrin antagonists such as small molecules (ATN-161, JSM6427) or function-blocking antibodies (volociximab, OS2966) have been considered as potential therapeutic approaches [32].

### 2.1. β1 Integrins Promote Resistance to EGFR-Targeted Therapies

#### 2.1.1. Cooperation between β1 Integrin and EGFR in Cancer Cells

EGFR was the first growth factor receptor reported as being activated in normal cells by β1 integrin adhesion to fibronectin, with or without the presence of EGF [61,62]. In cancer cells, β1 integrin potentiates EGF-mediated EGFR autophosphorylation in vitro and in vivo [38]. β1 integrin also regulates EGFR membrane trafficking and so modulates its oncogenic signaling activity [63]. In human ovarian carcinoma cells, α5β1 coordinates EGFR recycling to the plasma membrane in a way that enhances EGFR-Tyr845 phosphorylation and the serine kinase Akt downstream pathway, thus promoting cell invasion [64]. In lung carcinoma cells, the level of β1 integrin expression regulates the cell surface expression of EGFR and sustains its endocytic pathway [38]. It should be noted that although the literature has mostly described β1 integrins as positive regulators of EGFR, the relationship between integrin and EGFR appears to be far more complex. For instance, α5β1 has been described as restricting EGFR membrane localization and responsiveness to EGF stimulation [65], while α1β1 inhibits EGFR signaling by activating the protein phosphatase TCPTP (T-cell protein tyrosine phosphatase) [66]. β1 integrin/EGFR interaction was initially suggested by co-immunoprecipitation and confocal experiments, and FRET analysis revealed potential direct physical interaction between β1 integrin and either EGFR [59,67] or HER2 (ErbB2) [68]. Studies on β1 integrin cooperation with EGFR have revealed new avenues for improving the effectiveness of radiotherapy. Indeed, EGFR/β1 complex formation is a prognostic factor for radiotherapy resistance in glioma [59]. The importance of EGFR/β1 integrin cooperation in radiotherapy resistance has been confirmed by experiments which have shown that co-targeting the two of them radiosensitizes cancer cells [10,53]. Whole exome analysis has identified mTOR and KEAP1 as potential genetic biomarkers and molecular targets for radiosensitizing HNSCC [69]. By contrast, concomitant inhibition of β1 integrin and EGFR in HNSCC spheroids [70] and colon carcinoma [71] does not improve radiotherapy efficacy.

#### 2.1.2. Molecular Mechanism of β1 Integrin-Mediated Resistance to EGFR-Targeted Therapies

**Figure 1:**
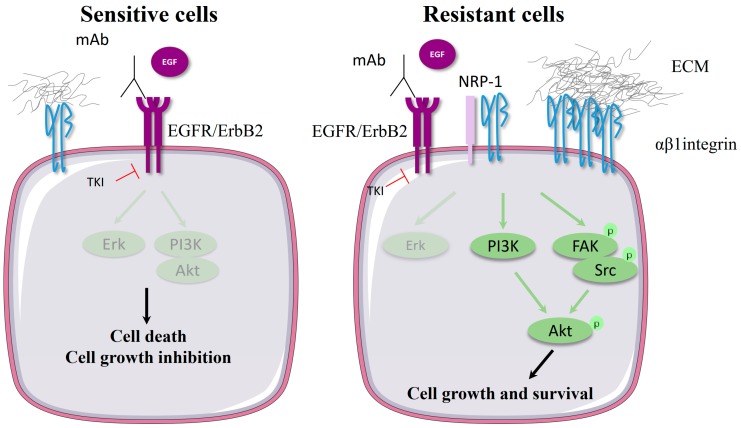
β1 integrin induces EGFR- or HER (ErbB2)-targeted therapy resistance. In sensitive cells, the inhibition of the ErbB receptor family by either antibodies or tyrosine kinase inhibitors (TKIs) blocks Erk and Akt pathway activation leading to cell death and cell growth inhibition. In resistant cells, β1 integrin or its associated extracellular matrix (ECM) proteins are often overexpressed, leading to the activation of β1-downstream pathways such as PI3K or FAK/Src. These pathways converge to activate the serine kinase Akt that promotes cell survival and cell growth. Alternatively, β1 integrin can be activated by coreceptors such as neuropilin-1 (NRP-1) to trigger EGFR-targeted therapy resistance independently of integrin-mediated cell adhesion.

The most common type of lung cancer is non-small cell lung carcinoma (NSCLC), and it is characterized by EGFR overexpression. Several oral tyrosine kinase inhibitors (TKIs) targeting EGFR are used in clinical practice for treating NSCLC, including gefitinib, erlotinib, afatinib, dacomitinib and osimertinib. These drugs show some efficacy, but NSCLC eventually relapses. Resistance to treatment is caused either by T790M EGFR mutation, which impedes TKIs (except osimertinib) from binding to EGFR by increasing its affinity to ATP, or by the activation of alternative or downstream signaling pathways [72]. Several groups independently report that acquired resistance to gefitinib has correlated with β1 integrin overexpression in NSCLC cells [13,73,74] or in lung tumor samples from patients refractory to gefitinib or erlotinib [13]. Interestingly, these studies revealed that β1 integrin overexpression occurs in NSCLC cells that do or do not harbor EGFR T790M mutations, irrespective of EGFR-phosphorylation level. Both antibody-mediated functional inhibition and siRNA-mediated silencing of β1 integrin sensitize NSCLC to TKIs in vitro and in vivo [13,38,73], demonstrating that β1 integrin is instrumental in TKI resistance. Conversely, the vector-mediated overexpression of β1 integrin protects cancer cells from TKI-induced cell growth inhibition [14,38]. Downstream of the β1/PI3K and β1/Src/FAK pathways, the serine kinase Akt plays a pivotal role in resistance to gefitinib or erlotinib (Figure 1) [13,38,73,75]. FAK is an essential protein in integrin/growth factor receptor crosstalk and could be a valuable target for sensitizing cancer cells to TKIs [76]. Integrin-dependent FAK activation decreased cancer cells’ sensitivity to anti-EGFR drugs [10,14]. A series of studies confirmed the importance of FAK signaling in resistance to first- (erlotinib), second- (afatinib) and third-generation (osimertinib) EGFR TKIs [28,29,30,31].

β1 integrins can also trigger resistance to antibody-mediated EGFR inhibition. In colon carcinoma cells, the fibronectin/α5β1 axis overcomes the inhibition of EGFR-mediated cell growth by mAb225, the murine form of the chimeric anti-EGFR antibody cetuximab [12]. In lung carcinoma cells, cetuximab activates the p38/ATF2 pathway. This enhances fibronectin synthesis, which in turn dampens cetuximab’s cytotoxic effect both in vitro and in xenografted mice. α5β1 integrin-silencing sensitized NSCLC cells to cetuximab monotherapy, showing that α5β1 integrin-mediated adhesion to fibronectin plays an essential role in reducing cetuximab activity in lung carcinoma cells [10,53]. In pancreatic ductal adenocarcinoma cells, the overexpression of β1 activates the FAK/Src/Akt pathway, triggering EGFR ligand-independent cell growth and thus bypassing cetuximab antagonist activity [11].

### 2.2. B1 Integrins Promote Resistance to Therapies Targeting HER2

HER2 (ErbB2) is another member of the EGFR family with intrinsic tyrosine kinase activity and is devoid of a ligand-binding domain. Overexpressed in nearly 30% of breast cancers, HER2 plays an important role in cancer progression. It is a highly attractive target for treatment with trastuzumab or pertuzumab, two humanized HER2− targeted antibodies, or for treatment with lapatinib, a TKI. Despite the radical improvement in the prognosis of HER2+ breast cancer brought about by these drugs, most patients with HER2+ tumors relapse and progress within a few years [77]. Using a genetic approach in mice, β1 integrin expression has been shown to play a critical role in HER2− induced breast tumor progression but is not required for tumor formation [78]. An immunochemical analysis of clinical samples revealed that β1 integrin overexpression is a negative prognostic factor for patients with HER2+ breast cancer being treated with trastuzumab [68]. In vitro, β1 integrin is overexpressed in HER2+ breast tumor cells with de novo resistance to trastuzumab. β1 integrin knockdown by siRNA silencing or inhibition by function-blocking antibody therapy enhanced drug efficacy by inhibiting the Erk1,2 and Akt pathways [79]. In contrast, another report showed that HER2+ breast cancer cell lines with de novo resistance to trastuzumab were not sensitized by β1 integrin inhibition, presumably because they maintain HER2 hyperphosphorylation. However, in the same study, β1 integrin was shown to promote resistance to lapatinib/trastuzumab treatment via an upregulation of FAK and Src. In that setting, antibody-mediated inhibition of β1 integrin decreased the 3D-growth and survival of the resistant cells being treated [15]. HER3, a kinase-dead member of the EGFR family, regulates HER2 signaling by initiating ligand-induced HER2 activation in the HER2-HER3 heterodimer. Co-targeting HER3 (via siRNA-mediated silencing) and β1 integrin (via a function-blocking antibody) is more effective in controlling tumor growth in mice than the dual inhibition of HER2 (lapatinib) and β1 integrin (antibody) [80].

The tumor microenvironment may markedly affect the response to HER2− targeted therapy [3]. Laminin-332, a ligand of integrins α6β4, α6β1 and α3β1, is lost during the malignant transformation of breast cancer but remains expressed by normal tissue and may thus support the initial transition to invasive cancer. Integrin-dependent adhesion to laminin-332 elicits lapatinib and trastuzumab resistance in HER2+ human breast tumor cell lines [25]. Recently, Hanker and colleagues used genetic engineering of HER2+/PIK3CA^H1047R^ mice to generate tumors resistant to TPB treatment (trastuzumab + pertuzumab + burparlisib, a PI3K inhibitor). Whole genome sequencing did not reveal any acquired mutation that could explain the acquired resistance to TPB. RNA-seq analysis did reveal the upregulation of several ECM genes, including Col2a1, which codes for the collagen type II alpha 1 chain. Collagen II activates the β1 integrin/Src pathway, promoting tumor resistance to TPB. In clinical settings, collagen II expression on immunohistochemical analysis correlates with a poor response to HER2− targeted therapies [16]. Antibody-drug conjugates (ADCs) are a promising novel class of therapeutic agents that combine a cytotoxic agent with the antigenic selectivity of an antibody. Ado-trastuzumab emtansine (T-DM1) is an ADC consisting of trastuzumab and DM1, a microtubule inhibitor [81]. Despite a good initial response to the drug, most patients eventually relapse due to acquired resistance. Recent reports have documented alterations in the ECM/integrin pathway in T-DM1-resistant cancer cells [82,83], which represent promising new approaches to enhancing T-DM1 toxicity against cancer cells.

### 2.3. β1 Integrin Expression Confers Resistance to Anti-Angiogenic Therapies Targeting VEGFR or c-Met

Tumor neo-angiogenesis is the formation of new blood vessels from those pre-existing in the tumor. Neo-angiogenesis is a critical step in tumor progression as it enhances tumor growth and cancer cell metastasis. The concept of anti-angiogenic therapy, i.e., inhibiting pro-angiogenic factors, has remained disappointing, in part due to acquired resistance [84]. The role of integrin in endothelial cell migration and survival and in angiogenesis has been widely described [85]. Several reports indicate that β1 integrin plays a part in anti-angiogenic therapy resistance [35,86]. Bevacizumab, a humanized antibody against VEGF-A, was the first anti-angiogenic drug used in clinical practice [87]. Micro-array analysis revealed that a subset of bevacizumab-resistant glioblastomas (GBMs) harbor α5 integrin and fibronectin overexpression [88], likely due to hypoxia provoked by bevacizumab treatment [17]. The inhibition of β1 integrin could become a treatment avenue in the future, as β1 integrin knockdown in bevacizumab-resistant glioma cells prevents in vivo growth while OS2966, a β1 function-blocking antibody, potentiates bevacizumab therapy [17].

The hepatocyte growth factor (HGF)/c-Met pathway plays an important role in tumor angiogenesis as well as in the development of resistance to VEGFR inhibition by TKIs [89]. β1 integrin and c-Met are able to form a complex and drive mutual ligand-independent cross-activation [18,90], indicating that β1 integrin and c-Met crosstalk may represent an adaptive mechanism to anti-angiogenic therapies. C-Met and β1 integrin membrane trafficking are closely related. In vascular endothelial cells, HGF stimulates β1 integrin recycling to promote cell spreading, focal adhesion formation, cell migration and tumor angiogenesis [91]. Moreover, c-Met can induce β1 integrin endocytosis [92], and integrin can transmit cell survival signaling from early endosomes [93]. The serine–threonine kinase MAP4K4 activates β1 integrin and mediates the accumulation of activated c-Met in cytosolic vesicles [94]. Thus, β1 integrin/c-Met ligand-independent cooperation is not restricted to the cell surface and can occur in autophagy-like endosomal compartments [95]. Because VEGFR-2 activation sequesters β1 integrin from c-Met in patients, the β1 integrin/c-Met complex is associated with bevacizumab resistance in GBM. It is interesting to note that OS2966 can reduce the formation of the β1 integrin/c-Met complex [18], which could explain its anti-angiogenic activity in bevacizumab-resistant tumors [17]. Targeting β1 integrin/c-Met complex formation may open up new treatment options for overriding resistance to targeted therapy and so limiting tumor angiogenesis as well as c-Met-mediated cell growth [75].

**Figure 2:**
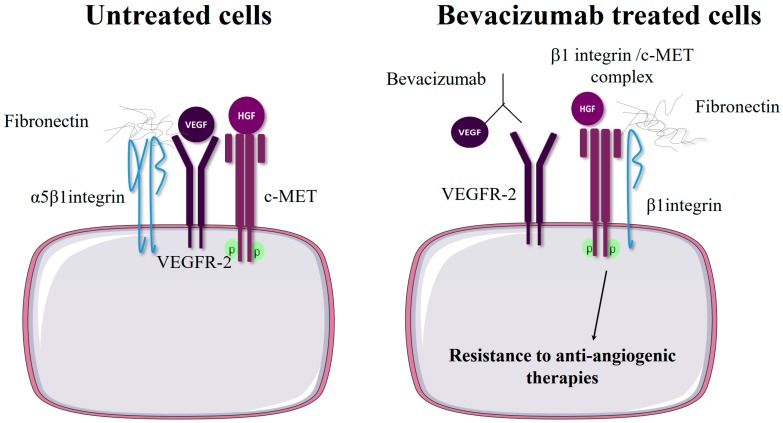
Hypothetical model presenting how β1/c-MET molecular complexes provide cancer cell resistance to anti-angiogenic therapies. In untreated cells, ligand-activated VEGFR-2 engages both α5β1 integrin and c-MET, impeding their physical contact. In α5β1 integrin-expressing cells, anti-angiogenic therapeutic intervention with bevacizumab decreases VEGF/VEGFR-2 binding. β1/c-MET complex formation is thus promoted, which leads to the cross-activation of both receptors and the activation of the downstream AKT signaling pathway (adapted from [18]).

It seems clear that β1 integrins play a pivotal role in resistance to RTK-targeted therapies both in vitro and in vivo. The pharmacological manipulation of integrins [14,15,18,79,80] or downstream signaling molecules such as FAK or Akt [51,52,53,54,58,59,60,61] has shown some efficacy in preclinical models and may offer promising new avenues to sensitizing cancer cells to anti-RTK therapies. β1 integrin expression levels could also represent a potent biomarker for stratifying patients likely to derive greater benefit from anti-RTK therapy, but the search for a molecular complex such as β1/EGFR, β1/HER2 or β1/c-Met could lead to even more promising strategies (Figure 2) [18,59,68].

In solid tumors, resistance to targeted therapies can be mediated by β1 integrin via a wide diversity of mechanisms that may require ligand-dependent or -independent integrin functions, or β1 integrin interaction with RTKs or with other co-receptors. The clinical relevance of the in vitro and vivo studies was mainly evident in glioma and breast cancers. Even with the promising therapeutic role of β1 integrin, it is important to keep in mind the complexity of integrin functions and the fact that the α subunits involved in the process remain indeterminate most of the time, although we know their importance in integrin function. The present data could also benefit from a patient stratification, allowing decreased doses of targeted therapy and consequently fewer secondary effects.

## 3. αvβ Integrins

αvβ integrins are a large family of integrins (αvβ3, αvβ5, αvβ6 and αvβ8). αv integrins drive cancer progression and are upregulated either by cancer cells or endothelial cells in many solid tumors, including colon carcinoma, melanoma, and breast, lung, pancreatic and brain cancers. αvβ integrins are characterized by their capacity to recognize the RGD (arginine-glycine-aspartate) peptide sequence in a large variety of ligands (such as vitronectin, fibronectin and osteopontin) [96]. αvβ integrin expression and activation drive the intracellular signaling that promotes cancer cell survival, invasion, metastasis, angiogenesis, and self-renewal [5,97], as well as chemotherapy resistance [98,99] and radiotherapy resistance [100,101,102,103,104]. Extensive preclinical studies have established αvβ3 inhibitors as potential new therapeutic tools [103,105,106,107], with several trials evaluating their efficacy in clinical settings as a result [108,109,110]. Cilengitide (EMD121974, Merck), a cyclic pentapeptide derived from the RGD sequence, was the most promising drug and was evaluated in clinical trials in newly diagnosed GBM. Unfortunately, these trials revealed that cilengitide did not improve the outcomes of patients receiving chemo- and radiotherapy [111,112,113], making it necessary to re-evaluate the treatment conditions or improve the molecular-based selection of patients who could benefit from cilengitide. Recently, Cosset and colleagues have shown that in GBM, αvβ3 integrin enhances the expression of the high-affinity glucose transporter GLUT3 via PAK4 (P21 Activated Kinase 4)/YAP (Yes-associated protein) pathway activation. The overexpression of GLUT3 increased tumor cell survival in a glucose-depleted environment. Furthermore, using genomic analysis the authors identified a subset of αvβ3/GLUT3-expressing tumors that were addicted to GLUT3 as well as highly sensitive to cilengitide and function-blocking anti-αv antibodies [114].

### 3.1. αv Integrin Triggers Resistance to Anti-EGFR Therapies

**Figure 3:**
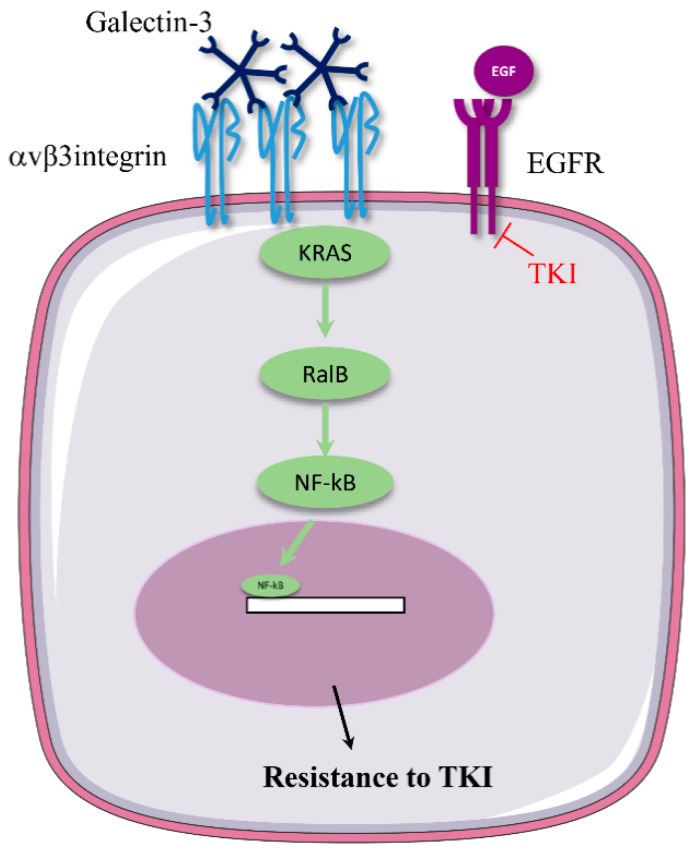
β3/KRAS/RalB/NFkB pathway mediates EGFR-targeted therapy resistance. In EGFR TKI-treated tumors, cells overexpressing αvβ3 integrin are selected, leading to a resistant tumor. By binding to oligosaccharide moieties of β3 integrin, galectin-3 promotes integrin/KRAS interaction independently of integrin-mediated adhesion to ECM proteins. KRAS activates the downstream RalB/NFkB pathway that leads to therapy resistance by promoting a stem cell-like phenotype (adapted from [19]).

The work of Seguin and colleagues paved the way for the demonstration of a pivotal role for αvβ3 integrin in resistance to EGFR-targeted therapy [19]. They first established that αvβ3 integrin was selectively expressed by tumor-initiating cells from lung and pancreatic carcinoma patient-derived-xenografts (PDXs). More strikingly, β3 expression drove lung and pancreatic cancer cell resistance to TKIs targeting EGFR (erlotinib and lapatinib) both in vitro and in mice. Conversely, the short hairpin RNA-mediated depletion of β3 sensitized cells to the TKIs. In fact, TKI treatment of human PDX tumors led to the selection of β3-positive cells that acquired stem cell-like and resistant phenotypes. Mechanistically, β3 integrin activates the KRAS (V-Ki-ras2 Kirsten rat sarcoma viral oncogene homolog)/RalB (Ras-like proto-oncogene B)/NF-kB pathway. It is important to note that the activation of this pathway is independent of the canonical FAK pathway and of integrin/ECM interaction. While this is surprising at first glance, the same group had already observed that αvβ3 integrin could promote tumor progression independently of ligand binding and FAK activation [115]. The authors discovered that the recruitment of KRAS and the consequent activation of RaIB by β3 required β3 binding to galectin-3, a cell adhesion protein with a specific affinity for β-galactoside glycoconjugates (Figure 3). β3 integrin may also play a pivotal role in mutant KRAS oncogenic function [116]. In a subset of lung and pancreatic adenocarcinomas addicted to mutant KRAS, the disruption of galectin-3/β3 interaction by GCS-100, a galectin-3 antagonist currently under clinical development [117], released mutant KRAS from β3 and inhibited tumor growth in mice. While these results may suggest that GCS-100 could sensitize lung cancer cells to TKIs, the authors have not yet explored this possibility. A growing body of data indicates that microRNA (miRNA) dysregulation modulates gefitinib resistance in lung carcinoma [118,119,120,121,122,123,124] In gefitinib-resistant cells, a miRNA targeting the 3′-UTR of β3 integrin (miR-483-3p) is silenced by epigenetic methylation. The forced overexpression of β3 integrin can restore gefitinib resistance in miR-483-3p-expressing cells through the activation of a β3/FAK/ERK pathway and epithelial to mesenchymal transition induction [20].

The role of αv integrin in resistance to anti-EGFR therapy has been assessed in clinical settings. Cilengitide has been evaluated in combination with cetuximab in two phase II clinical trials on HNSCC and NSCLC [125]. Cilengitide did not improve patient outcomes. However, ex vivo experiments on patient-derived samples showed that a subset of sensitive tumors could be selected based on the inhibition of colony formation or cytokine release [126,127]. The phase I/II Poseidon trial explored the efficacy of a combination treatment using abituzumab, an αv integrin inhibitor, and cetuximab in KRAS wild-type metastatic colorectal cancer. Again, the trial did not reach the primary phase II endpoint, but the authors did observe that patients overexpressing αvβ6 integrin might benefit from the abituzumab/cetuximab plus irinotecan combination treatment compared to cetuximab plus irinotecan alone [128]. In the future, therefore, reliable biomarkers may emerge for selecting patients likely to benefit from the synergy between αv integrin and EGFR inhibition.

### 3.2. αvβ3 Integrin Involvement in Resistance to Drugs Targeting Other RTKs

Insulin-like growth factors and their cognate receptors are important in cancer progression [129]. Antibody-based therapy against IGF-1R has shown limited efficacy in phase II/III clinical trials [130]. αvβ3 integrin enhances IGF-1R signaling activity through the joint ligand-dependent activation of both receptors. However, another model of crosstalk has been proposed in which the IGF-1R ligand, IGF-1, directly binds to the β3 integrin subunit and promotes the anchorage-independent formation of a β3/IGF-1/IGF-1R ternary complex [131,132,133,134]. αvβ3 integrin significantly contributes to resistance to IGF-1R-targeted TKIs [19]. In HNSCC and NSCLC, during treatment with cixutumumab, a humanized anti-IGF-1R antibody, the Src/Akt pathway is activated by IGF-1/β3 integrin interaction independently of cell/ECM interaction. The molecular targeting of β3 integrin increased cixutumumab’s efficacy both in vitro and in mice [21]. While these preclinical data are encouraging, the role of the β3/Src pathway in resistance to anti-IGFR treatment has not yet been evaluated in clinical settings.

Sorafenib is a multikinase inhibitor for treating hepatocellular, kidney and thyroid carcinomas [135]. According to KINOMEscan data from the Library of Integrated Network-based Cellular Signatures project (http://lincs.hms.harvard.edu/), among the numerous kinases inhibited by sorafenib are the receptors for PDGF, VEGF and fibroblast growth factor. In acute myeloid leukaemia, αvβ3 integrin expression is a negative prognostic factor and is associated with a decrease in sorafenib activity. Mechanistically, αvβ3 integrin is activated by osteopontin and stimulates the PI3K/Akt/GSK3 pathway [22]. In hepatocellular carcinoma, galectin-1, a β-galactoside-binding protein, is a negative prognostic factor [136], whose expression increases sorafenib resistance [23]. Galectin-1 stimulates αvβ3 expression and hyperactivation of the β3/FAK/PI3K/Akt pathway to potentiate EMT, but a clear demonstration of a role of β3 in resistance to sorafenib is missing in this study [23]. Galectin-1 has been shown to interact with other integrins, including β1 [136,137,138]. Thus, given their ability to regulate both β1 and β3 integrin function, dysregulation of galectin-1 and galectin-3 expression in the tumor microenvironment may have a profound impact on the efficacy of therapies targeting RTKs.

In vitro and vivo data revealed that αvβ3 integrin may support resistance to therapies targeting several RTKs (EGFR, IGFR, PDGFR, FGFR, VEGFR). Furthermore, mechanisms of resistance to EGFR and IGFR TKIs have been identified and found to be independent of αvβ3 binding to ECM ligands, via recognition of the RGD sequence [19,21]. Given the clinical failure of cilengitide to improve the outcomes of cetuximab-treated patients [122,123], the time may have come for the development and use of integrin-targeted drugs that do not target integrin binding to ECM proteins such as RGD-derived peptide.

## 4. α6β4 Integrins

As a nucleator of hemidesmosomes, α6β4 integrin, a laminin-332 (also named laminin-5) receptor, is a master regulator of epithelium integrity and homeostasis. Hemidesmosomes are junctional structures that mediate the firm adhesion of epithelial cells to the basement membrane by linking intermediate filaments to laminin-332. Dysregulation of α6β4 leads to aberrant hemidesmosomal and epithelial dysfunction [139,140]. It has been reported that hemidesmosomal α6β4 integrin is not fully competent for signal transduction, suggesting that epithelium/basement membrane attachment remains its main function in healthy tissue [24,141].

### 4.1. Crosstalk between α6β4 Integrin and Growth Factor Receptors

Hemidesmosomes are dynamic adhesive structures that must be dismantled to allow epithelial cell migration during wound healing. α6β4 interaction with hemidesmosomal proteins is tightly regulated by EGFR signaling pathways [142,143,144,145,146,147]. EGFR activation promotes the phosphorylation of serine residues in the signaling domain of β4, which disrupts its interaction with plectin, a linker between integrin and intermediate filaments. The phosphorylated β4 cytoplasmic domain serves as a docking platform to stimulate signaling pathways (such as Src, PI3K, Rho GTPases) and synergize with RTKs. The clinical significance and roles of α6β4 in carcinoma development and progression have been extensively reviewed [148,149]. In mice, the ablation of α6 integrin in intestinal epithelial cells has led to hemidesmosomal disruption and a loss of epithelial/basement membrane junction integrity. These mice spontaneously developed long-standing colitis and subsequent colorectal carcinoma [150]. This may suggest that α6β4 acts as a tumor suppression gene. However, except in basal carcinoma and prostate carcinoma, α6β4 is overexpressed in epithelial tumors and largely contributes to cancer progression and poor prognosis [148]. In these tumors, over-activation of the EGFR, HER2 or c-Met pathways disrupted plectin/α6β4 integrin coupling and hemidesmosomal disassembly [151,152]. α6β4 becomes fully competent for signal transduction and cooperation with RTKs [24,153], and can promote cancer cell proliferation and survival, tumor invasion, metastasis and angiogenesis [149].

### 4.2. α6β4 Integrin and Resistance to Anti-HER2 Therapies

Although α6β4 integrin is a pertinent therapeutic target in most forms of carcinoma, few studies have evaluated its potential to trigger RTK-targeted therapy resistance. Using an in vitro knock-in system, Guo and colleagues established a murine model in which endogenous β4 integrin was replaced by signaling-defective β4 integrin (lacking the carboxyterminal moiety of its intracellular domain) in the mammary gland of MMTV-Neu(YD) mice [24]. In this model, wild-type (WT) β4 integrin, but not the mutant form, synergized with HER2 to increase mammary carcinoma tumorigenicity. Interestingly, the therapeutic activity of gefitinib was dampened in WT-β4 mice compared to mutant-β4 mice, indicating that β4 signaling function can promote resistance to anti-HER2 drugs. The molecular pathway eliciting this resistance is independent of HER2 phosphorylation and remains unknown. Small molecules or antibodies capable of disrupting the integrin/HER2 heterocomplex may improve HER2-targeted therapies. In human breast cancer cells, the expression of laminin-332 or α6β4 integrin triggers a notable resistance to trastuzumab and lapatinib [25]. Gefitinib-mediated cell toxicity was substantially reduced when hepatocarcinoma cells were exposed to laminin-332 but not to other ECM proteins such as collagen or fibronectin [27]. More recently, high β4 integrin expression was associated with a gefitinib-resistant phenotype in gastric cancer cells [26]. The resistant phenotype could be reverted by RNA-mediated β4 silencing, whereas sensitive cells became more resistant to gefitinib after β4 overexpression. A clinical study in 38 patients has indicated some correlation between β4 expression and gefitinib resistance. However, given the small sample size, it is far too early to draw any conclusion about the potential repercussions of this observation [26]. Another clinical study showed that β4 integrin polymorphism expression was associated with resistance to therapy. The authors examined the expression level of three different β4 polymorphisms in HER3-negative/KRAS WT metastatic colorectal cancer from patients receiving irinotecan/cetuximab. Although conducted in a small cohort of patients, the study showed a significant decrease in progression-free survival and overall survival in patients harboring the β4 rs8669 G polymorphism [154].

It is clear that α6β4 can unleash the oncogenic potency of RTKs in cancer cells. Data obtained from cell lines, murine models and patient samples are the first insight into the role of α6β4 integrin in resistance mechanisms to TKIs and antibodies against members of the HER family. Further investigation is required to assess the clinical relevance of these observations. Targeting α6β4 integrin/RTK interaction could be a promising strategy for overcoming resistance. Another strategy could be to use integrin β4 expression and polymorphism to stratify patients to EGFR-targeted therapies.

## 5. Integrins and Carcinoma-Associated Fibroblasts

Tumor progression relies on close interaction and communication between cancer cells and cancer-associated fibroblasts (CAFs) through several mechanisms, including paracrine signals (transforming growth factor-β, IGF-1, exosomes), cell-to-cell contact and ECM remodeling [155,156]. Emerging data indicating that CAFs can decrease therapeutic response (including to anti-RTK drugs) have been extensively reviewed [157]. We will restrict our analysis to research incriminating integrin involvement in CAF-mediated therapy resistance.

In breast cancer cells, physical interaction between cancer cells and stromal cells (mesenchymal stem cells or CAFs) strongly protects against lapatinib or trastuzumab [158,159]. In those two studies, no experimental data could attest to any role for integrin in CAF-mediated resistance. However, CAF/breast cancer cell interaction requires the synthesis of hyaluronic acid by CAFs [159]. Hyaluronic acid can bind to and activate CD44, a known partner and regulator of integrins [160]. Alternatively, integrin may also be involved in CAF/cancer cell interaction. For instance, we showed that α5β1 integrin can promote cell/cell interaction during tumor spheroid formation [161]. This particular integrin can mediate CAF interaction with a highly aggressive subset of ovarian carcinoma cells. Heterotypic CAF/cancer cell spheroids promote the metastasis of ovarian cells in mice [162].

In vitro assays have revealed that collagen fiber synthesis and assembly by CAFs promote lung cancer cell resistance to gefitinib and osimertinib [163]. Another study showed that collagen-mediated resistance to TKIs requires the activation of the Akt/mTOR pathway [164]. Interestingly, the inhibition of collagen synthesis or β1 integrin function suppresses this resistance, offering new therapeutic options [163]. These observations may be clinically relevant as increased collagen deposition has been observed in erlotinib-resistant xenografts [163], and as progression-free survival has been seen to decrease in gefitinib-treated patients with collagen-rich lung tumors [164]. Another group used genetically modified mice expressing inducible RAF-driven tumors to model melanoma development in the ear. For the longitudinal monitoring of tumor development, the authors used intravital two-photon microscopy of fluorescently tagged melanoma cells. Upon MEK inhibition, the tumors transiently responded but returned to their original size after 12 weeks of treatment. It was noted that the cells that survived MEK inhibition co-localized with collagen bundles (imaged by second harmonic generation) [165].

**Figure 4:**
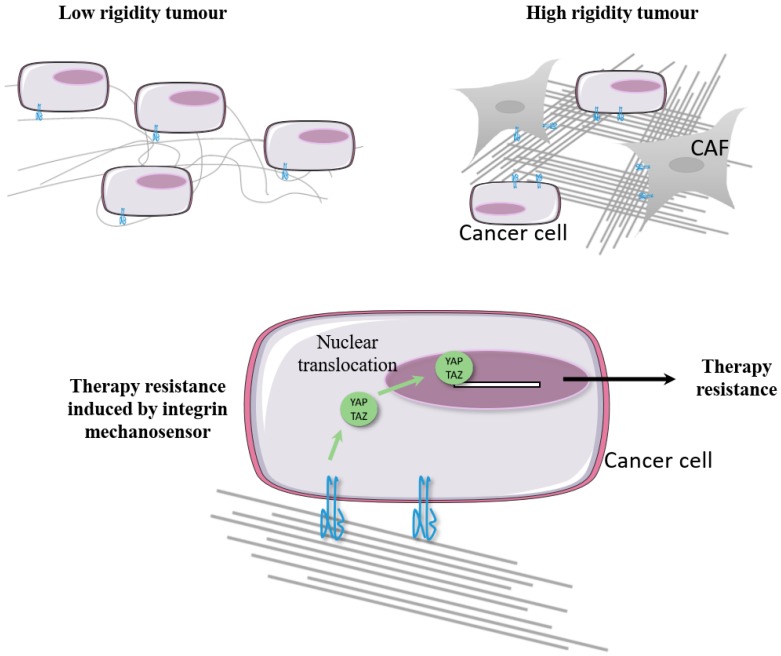
Hypothetic model showing how integrins may trigger therapy resistance in stiff micro-environmental niches. Therapy-resistant tumors are often characterized by an increase in matrix stiffness. Cancer-associated fibroblast (CAF) integrins (mainly α5β1 integrin) generate mechanical forces that increase ECM protein assembly and matrix rigidity. In cancer cells, the sensing of CAF-generated tension by integrins activates transcriptional co-regulators YAP and TAZ and their translocation to the nucleus. The transcriptional response leads to therapy resistance (adapted from [186]).

Hirata and colleagues have shown that melanoma-associated fibroblasts can drive resistance to the BRAF (v-Raf murine sarcoma viral oncogene homolog B) inhibitor vemurafenib by stimulating fibronectin production and remodeling, and subsequently promoting β1/Src/FAK pathway signaling in melanoma [166]. Another study confirmed the crucial role of fibronectin/β1 integrin signaling in melanoma adaptation to BRAF inhibition [167]. In both studies, following vemurafenib treatment, increases in fibrous ECM were observed in xenograft tumors and in several excised human melanomas. The concomitant inhibition of BRAF and FAK to suppress PDX growth in mice has been advanced as one way of improving therapy [166]. The efficacy of this therapeutic option was recently confirmed through the screening of a kinase inhibitor library [168]. Matrix stiffening generates mechanical forces that are transduced through the plasma membrane by integrin adhesome and stimulate YAP and TAZ (transcriptional coactivator with PDZ-binding motif) nuclear translocation and activation [169,170]. Cancer cells that express the activating mutant of Ras (RASG12D) are addicted to this oncogene. Studies from two different laboratories conjointly established that YAP and TAZ activation drive mutant KRAS-independent tumor growth and progression [171,172]. Therefore, increased matrix stiffness is sufficient to protect BRAF-mutant melanoma cells from BRAF inhibition [166], while YAP/TAZ activation induces resistance to therapy targeting the RAS/RAF pathway [173,174]. Additionally, several concurrent reports confirmed that matrix stiffening modulates cancer cell response to TKIs [175,176,177]. As mechanotransducers, integrin and FAK play key functions in tension generation by CAFs [178,179,180,181,182]. In turn, ECM stiffening enhances integrin signaling in cancer cells [183] and contributes to cancer progression [184]. Hence integrin mechanosensing plays multiple roles in the microenvironment (both in stromal and cancer cells) that promote tumor growth and therapy resistance [185,186]. It can be hypothesized that YAP/TAZ regulation by integrin mechanotransduction provides a safe haven that protects cells from therapies targeting the RTK pathway (Figure 4).

## 6. Conclusions

As shown in this review, integrin interacts with several RTKs such as the HER family, c-Met, PDGFR and others. These interactions can give cells an intrinsic ability to better adapt to and resist targeted therapies. Several integrin inhibitors were described and are being tested in clinical settings, albeit with no strong benefit, not even in combination with RTK inhibitors. It should be noted that, although in clinical practice integrin-targeted therapies are based on the inhibition of their adhesive function by small antagonist molecules or monoclonal antibodies, several studies have shown that integrin/RTK interactions and integrin-mediated resistance to therapies targeting RTK can be elicited by unbound integrins [19,95,115,153]. The ability of integrins to form functional molecular complexes with RTKs makes the situation much more difficult to understand. But it also makes new treatment approaches possible, be it predicting the efficacy of anti-RTK therapies in subpopulations of patients based on their level of heterocomplex expression [18,59] or developing treatments for disrupting integrin/RTK complex formation [17,18]. Aptamers, small nucleic acids used in treatment [187], can disrupt EGFR/β3 integrin interaction to inhibit tumor growth [188]. Meanwhile, targeting EGFR/uPAR using an integrin antagonist confers sensitivity to vemurafenib [189]. Finally, Kim and colleagues created an antibody that co-targets EGFR and neuropilin-1, a receptor that physically interacts with active β1 integrin. This antibody enhanced β1 integrin internalization and so led to the inhibition of β1 signaling, reducing tumor volume in in vivo experiments [11]. All these examples illustrate the strong potential of this new therapeutic concept.

New functions of integrins are continually being discovered, proving their importance in therapy resistance. A better understanding of the molecular mechanisms underlying the integrin/RTK relationship could one day make it possible to improve the efficacy of therapies targeting RTKs.

## Figures and Tables

**Table 1:** Cases of integrin-mediated resistance to RTK-targeted therapies cited in this review.

RTK	Therapies Targeting RTK	Type of Tumor	Experimental Model	Patient Data	Integrin Modulation	Mechanisms of Resistance	Ref
**β1 integrin**							
**EGFR**	Cetuximab	Head and neck squamous cell carcinoma	A549 cells	-	Cetuximab-induced fibronectin overexpression. siRNA-mediated depletion of β1 and α5	Cetuximab enhances p38/ATF2-dependent fibronectin production and the activation of the focal adhesion kinase (FAK)/Erk pathway. siRNA-mediated depletion of β1 and α5 integrin decreases the cell survival of cetuximab-treated cells.	[10]
**EGFR**	Cetuximab	Pancreatic cancer	Miapaca-2, Capan-2, SW1990 AsPC-1, BXPC-3, PANC-1	-	-Endogenous overexpression of β1 integrin in resistant cells -siRNA-mediated depletion of β1	β1 overexpression in resistant cells stimulates Src and Akt pathways. Extracellular matrix (ECM)-independent activation of β1 is mediated by its interaction with neuropilin-1. siRNA-mediated depletion of β1 or inhibition of β1/neuropilin-1 interaction increases cetuximab cell toxicity.	[11]
**EGFR**	mAb225	Colon cancer	Caco-2	-	Plasmid-induced α5 overexpression	Fibronectin stimulation of α5-expressing cells overrides mAb225-mediated cell growth inhibition. Integrin activates epidermal growth factor receptor (EGFR) kinase and the mitogen-activated protein kinase (MAPK) pathway.	[12]
**EGFR**	Gefitinib Erlotinib	Lung cancer	PC-9 and 11-18	Patient samples	-Endogenous overexpression of β1 integrin in resistant cells and tumors -siRNA-mediated depletion of β1	siRNA-mediated silencing of β1 restores Erlotinib potency to inhibit cell proliferation and the Src and Akt pathways.	[13]
**EGFR**	PD1530335 (AG1517)	Glioma	Glioma stem-like cells (GSCs) isolated from glioblastoma (GBM) surgical pieces	-	Lentiviral-mediated β1 overexpression	Delocalization of β1 integrin from lipid raft sensitizes GSC to tyrosine kinase inhibitor (TKI)-induced apoptosis. β1 overexpression protects GSC from apoptosis in a FAK-dependent manner.	[14]
**HER2**	Trastuzumab Lapatinib	Breast cancer	HER2+ cells (BT474, HCC1954)	-	-Endogenous overexpression of β1 integrin in resistant cells. -siRNA-mediated depletion of β1 and function-blocking mAb	Overexpression of β1 enhances FAK and Src phosphorylation. Silencing or functional inhibition of β1 integrin sensitizes cells to HER-2 inhibition (cell proliferation, apoptosis, clonogenic assays) in a FAK-dependent way.	[15]
**HER2**	TPB (trastuzumab + pertuzumab + burparlisib)	Breast cancer	Tumors cells derived from HER2+/PIK3CAH^1047R^ mice, MDA-MB453, HCC1954 cell lines	Patient samples and data	-Endogenous overexpression of collagen II in resistant tumors - β1 function-blocking mAb	Resistance to anti-HER2 tritherapy activates β1 integrin and Src pathways. Inhibition of β1/Src blocks coll II-induced resistance to TPB (cell growth, cell survival)	[16]
**VEGFR**	Bevacizumab	Glioma	U87, bevacizumab-resistant cell lines derived from surgical pieces (in vitro and xenografts)	Patient samples and data	-Endogenous overexpression of β1 integrin in resistant cells. -shRNA-mediated depletion of β1 and function-blocking mAb	Bevacizumab induces hypoxia that is associated with increased β1 and FAK expression. β1 inhibition (function-blocking mAb) results in increased cell apoptosis and in disrupted tumor mass formation in the treated tumor	[17]
**VEGFR**	Bevacizumab	Glioblastoma breast cancer	PDX for bevacizumab-resistant human GBM GBM and breast cancer cells	Patient samples	Increased β1/c-Met complex formation in bevacizumab-resistant tumors	Vascular endothelial growth factor receptor (VEGFR)-2 activation impedes β1/cMet complex formation. Resistance to antiangiogenic therapy increased β1/cMet complex formation and cross-activation of both receptors.	[18]
**β 3 integrin**							
**EGFR**	Erlotinib Lapatinib	Lung cancer	A549 and H23 xenograft	Patient samples	shRNA-mediated depletion of β3	EGFR TKI treatment induces selection of β3-positive cancer stem cells. Integrin β3 (in a ligand-independent way) interacts with galectin-3 to promote KRAS/RalB/NFkB activation, thereby promoting cell survival.	[19]
**IGFR**	Linsitinib	Pancreatic cancer	Panc-1 and FG xenograft	-			
**EGFR**	Gefitinib	Lung cancer	HCC827	-	-Epigenetic silencing of β3-targeting miR-489-3p in resistance cells -Lentivirus-mediated expression of β3 -Inhibitor or mimic of miR-489-3p	Hypermethylation of miR-483-3p in resistant cells activates the β3-dependent FAK/Erk pathway to promote cell survival and EMT	[20]
**IGFR**	Cixutumumab	Head and neck squamous cell carcinoma	Several cell lines	Patient samples	shRNA-mediated depletion of β3 and function-blocking mAb	Upon cixutumumab treatment, insulin-like growth factor (IGF)-1 directly binds to integrin ανβ3, increasing Src/Akt-dependent proliferation and survival.	[21]
		Lung cancer	686LN, UMNSCC38, H226B, A549 In vitro and xenograft	-			
**PDGFR, VEGFR, FGFR**	Sorafenib	Acute myeloid leukemia	MV4-11	Patient samples and data	-Endogenous overexpression of β3 integrin in resistant cells - β3 function-blocking mAb	Activation of β3/PI3K/Akt/GSK3β/β-catenin pathway reduces apoptotic level and increases cell proliferation in resistant cells	[22]
**PDGFR, VEGFR, FGFR**	Sorafenib	Hepatic cancer	Huh-7, Hep3B, SK-Hep-1, HepG2, PLC/PRF/5	-	-shRNA-mediated depletion of β3	Forced expression of galectin-1 elevates β3 expression and activates the FAK/PI3K/Akt pathway to trigger EMT. This is correlated with an increased resistance to sorafenib in galectin-1 expressing cells.	[23]
**β4 integrin**							
**HER2**	Gefitinib	Breast cancer	Murine model mammary gland MMTV-Neu (YD)	-	-Forced expression of β4 mutant (depleted from its signaling domain)	α6β4/ErbB2 complex activates transcription factor STAT3 and c-Jun to promote cancer progression. The signaling domain of β4 is required to trigger gefitinib resistance by an unknown mechanism, whereas ErbB2, C-Jun and STAT3 phosphorylation is still inhibited by gefitinib.	[24]
**HER2**	Trastuzumab Lapatinib	Breast cancer	BT474, ZR-75-1, SKBR3, MD-MB-453	-	shRNA-mediated depletion of α6β4 and function-blocking mAbs	Integrin-mediated adhesion to laminin-5 promotes resistance to anti-ERB2 therapies. Removal of CD151 (an integrin co-receptor) or FAK sensitizes cells to drugs (cell proliferation)	[25]
**EGFR**	Gefitinib	Gastric cancer	SGC7901	Patient samples	-Endogenous overexpression of α6β4 integrin in resistant cells -siRNA-mediated depletion of α6β4	Endogenous or forced expression of β4 integrin promotes gefitinib resistance (cell proliferation and apoptosis). β4 expression is correlated with a decrease in p-EGFR protein levels.	[26]
**EGFR**	Gefitinib	Hepatic cancer	HLF, Alexander, HepG2, Sk-Hep1	-	Laminin-332 expression	Lm-332-dependent activation of integrin dampens gefitinib effectiveness in cell proliferation survival and apoptotis assays. Lm-332 potentiates the activation of Akt in gefitinib-treated cells.	[27]
**FAK**							
**EGFR**	Erlotinib	Lung cancer	A549, H1299, H1975, HCC827, HCC4006 Xenograft of A549	-	FAK inhibitors	Combination of FAK inhibitors and erlotinib is more potent than a single agent to reduce cell viability (2D and 3D models), to increase the apoptosis pathway and cell cycle arrest in resistant cells, and to reduce tumor growth in vivo. The sensitization of erlotinib by FAK inhibitors is associated with a strong inhibition of Akt.	[28]
**EGFR**	Erlotinib Osimertinib	Lung cancer	PC-9, H1975, HCC827, HCC4006, H3255, 11-18 cell lines PC-9 xenografts	-	FAK inhibitor	Activation of FAK and Src family kinases (SFK) pathways attenuates the efficiency of EGFR therapies presumably via the sustained activation of MAPK and Akt pathways. Concomitant inhibition FAK, Src and EGFR inhibitors potently inhibit MAPK and Akt pathways and cell proliferation.	[29]
**EGFR**	Afatinib Erlotinib Osimertinib	Lung cancer	PC-9, HCC827 Established TKI-resistant cells	-	siRNA-mediated depletion of FAK and inhibitor	Compensatory activation of SFKs, FAK and Akt is observed in TKI- resistant cells. FAK inhibition increased afatinib efficacy to inhibit cell survival and cell migration.	[30]
**EGFR**	Erlotinib	Lung cancer	H1299, H1650 cell lines H358 cell line and xenograft	-	siRNA-mediated depletion of FAK and inhibitor (PF-562271)	Mass spectrometry analysis revealed an aberrant phosphorylation of FAK in erlotinib-resistant cells. Inhibition of FAK led to a decrease in cell survival in erlotinib-treated cells.	[31]

## References

[1] Hanahan D., Weinberg R.A. (2011). Hallmarks of cancer: The next generation. Cell.

[2] Paraiso K.H.T., Smalley K.S.M. (2013). Fibroblast-mediated drug resistance in cancer. Biochem. Pharmacol..

[3] Zoeller J.J., Bronson R.T., Selfors L.M., Mills G.B., Brugge J.S. (2017). Niche-localized tumor cells are protected from HER2-targeted therapy via upregulation of an anti-apoptotic program in vivo. NPJ Breast Cancer.

[4] Ivaska J., Heino J. (2011). Cooperation between integrins and growth factor receptors in signaling and endocytosis. Annu. Rev. Cell Dev. Biol..

[5] Desgrosellier J.S., Cheresh D.A. (2010). Integrins in cancer: Biological implications and therapeutic opportunities. Nat. Rev. Cancer.

[6] Barczyk M., Carracedo S., Gullberg D. (2010). Integrins. Cell Tissue Res..

[7] Xing Z., Chen H.C., Nowlen J.K., Taylor S.J., Shalloway D., Guan J.L. (1994). Direct interaction of v-Src with the focal adhesion kinase mediated by the Src SH2 domain. Mol. Biol. Cell.

[8] Paul M.K., Mukhopadhyay A.K. (2004). Tyrosine kinase—Role and significance in Cancer. Int. J. Med. Sci..

[9] Sangwan V., Park M. (2006). Receptor tyrosine kinases: Role in cancer progression. Curr. Oncol..

[10] Eke I., Storch K., Krause M., Cordes N. (2013). Cetuximab attenuates its cytotoxic and radiosensitizing potential by inducing fibronectin biosynthesis. Cancer Res..

[11] Kim Y.-J., Jung K., Baek D.-S., Hong S.-S., Kim Y.-S. (2017). Co-targeting of EGF receptor and neuropilin-1 overcomes cetuximab resistance in pancreatic ductal adenocarcinoma with integrin β1-driven Src-Akt bypass signaling. Oncogene.

[12] Kuwada S.K., Li X. (2000). Integrin α5/β1 Mediates fibronectin-dependent epithelial cell proliferation through epidermal growth factor receptor activation. Mol. Biol. Cell.

[13] Kanda R., Kawahara A., Watari K., Murakami Y., Sonoda K., Maeda M., Fujita H., Kage M., Uramoto H., Costa C. (2013). Erlotinib resistance in lung cancer cells mediated by integrin β1/Src/Akt-driven bypass signaling. Cancer Res..

[14] Srikanth M., Das S., Berns E.J., Kim J., Stupp S.I., Kessler J.A. (2013). Nanofiber-mediated inhibition of focal adhesion kinase sensitizes glioma stemlike cells to epidermal growth factor receptor inhibition. Neuro. Oncol..

[15] Huang C., Park C.C., Hilsenbeck S.G., Ward R., Rimawi M.F., Wang Y.-C., Shou J., Bissell M.J., Osborne C.K., Schiff R. (2011). β1 integrin mediates an alternative survival pathway in breast cancer cells resistant to lapatinib. Breast Cancer Res..

[16] Hanker A.B., Estrada M.V., Bianchini G., Moore P.D., Zhao J., Cheng F., Koch J.P., Gianni L., Tyson D.R., Sánchez V. (2017). Extracellular matrix/integrin signaling promotes resistance to combined inhibition of HER2 and PI3K in HER2+ breast cancer. Cancer Res.

[17] Carbonell W.S., DeLay M., Jahangiri A., Park C.C., Aghi M.K. (2013). β1 integrin targeting potentiates antiangiogenic therapy and inhibits the growth of bevacizumab-resistant glioblastoma. Cancer Res..

[18] Jahangiri A., Nguyen A., Chandra A., Sidorov M.K., Yagnik G., Rick J., Han S.W., Chen W., Flanigan P.M., Schneidman-Duhovny D. (2017). Cross-activating c-Met/β1 integrin complex drives metastasis and invasive resistance in cancer. Proc. Natl. Acad. Sci. USA.

[19] Seguin L., Kato S., Franovic A., Camargo M.F., Lesperance J., Elliott K.C., Yebra M., Mielgo A., Lowy A.M., Husain H. (2014). An integrin β3-KRAS-RalB complex drives tumour stemness and resistance to EGFR inhibition. Nat. Cell Biol..

[20] Yue J., Lv D., Wang C., Li L., Zhao Q., Chen H., Xu L. (2018). Epigenetic silencing of miR-483-3p promotes acquired gefitinib resistance and EMT in EGFR-mutant NSCLC by targeting integrin β3. Oncogene.

[21] Shin D.H., Lee H.-J., Min H.-Y., Choi S.P., Lee M.-S., Lee J.W., Johnson F.M., Mehta K., Lippman S.M., Glisson B.S. (2013). Combating resistance to anti-IGFR antibody by targeting the integrin β3-Src pathway. J. Natl. Cancer Inst..

[22] Yi H., Zeng D., Shen Z., Liao J., Wang X., Liu Y., Zhang X., Kong P. (2016). Integrin alphavbeta3 enhances β-catenin signaling in acute myeloid leukemia harboring Fms-like tyrosine kinase-3 internal tandem duplication mutations: Implications for microenvironment influence on sorafenib sensitivity. Oncotarget.

[23] Zhang P.-F., Li K.-S., Shen Y., Gao P.-T., Dong Z.-R., Cai J.-B., Zhang C., Huang X.-Y., Tian M.-X., Hu Z.-Q. (2016). Galectin-1 induces hepatocellular carcinoma EMT and sorafenib resistance by activating FAK/PI3K/AKT signaling. Cell Death Dis..

[24] Guo W., Pylayeva Y., Pepe A., Yoshioka T., Muller W.J., Inghirami G., Giancotti F.G. (2006). β4 integrin amplifies ErbB2 signaling to promote mammary tumorigenesis. Cell.

[25] Yang X.H., Flores L.M., Li Q., Zhou P., Xu F., Krop I.E., Hemler M.E. (2010). Disruption of laminin-integrin-CD151-focal adhesion kinase axis sensitizes breast cancer cells to ErbB2 antagonists. Cancer Res..

[26] Huafeng J., Deqing Z., Yong D., Yulian Z., Ailing H. (2018). A cross-talk between integrin β4 and epidermal growth factor receptor induces gefitinib chemoresistance to gastric cancer. Cancer Cell Int..

[27] Giannelli G., Azzariti A., Fransvea E., Porcelli L., Antonaci S., Paradiso A. (2004). Laminin-5 offsets the efficacy of gefitinib (‘Iressa’) in hepatocellular carcinoma cells. Br. J. Cancer.

[28] Howe G.A., Xiao B., Zhao H., Al-Zahrani K.N., Hasim M.S., Villeneuve J., Sekhon H.S., Goss G.D., Sabourin L.A., Dimitroulakos J. (2016). Focal adhesion kinase inhibitors in combination with erlotinib demonstrate enhanced anti-tumor activity in non-small cell lung cancer. PLoS ONE.

[29] Ichihara E., Westover D., Meador C.B., Yan Y., Bauer J.A., Lu P., Ye F., Kulick A., de Stanchina E., McEwen R. (2017). SFK/FAK signaling attenuates osimertinib efficacy in both drug-sensitive and drug-resistant models of EGFR-mutant lung cancer. Cancer Res..

[30] Murakami Y., Sonoda K., Abe H., Watari K., Kusakabe D., Azuma K., Kawahara A., Akiba J., Oneyama C., Pachter J.A. (2017). The activation of SRC family kinases and focal adhesion kinase with the loss of the amplified, mutated EGFR gene contributes to the resistance to afatinib, erlotinib and osimertinib in human lung cancer cells. Oncotarget.

[31] Solanki H.S., Raja R., Zhavoronkov A., Ozerov I.V., Artemov A.V., Advani J., Radhakrishnan A., Babu N., Puttamallesh V.N., Syed N. (2018). Targeting focal adhesion kinase overcomes erlotinib resistance in smoke induced lung cancer by altering phosphorylation of epidermal growth factor receptor. Oncoscience.

[32] Barkan D., Chambers A.F. (2011). β1-Integrin: A potential therapeutic target in the battle against cancer recurrence. Clin. Cancer Res..

[33] Cordes D.N., Park C.C. (2007). beta1 integrin as a molecular therapeutic target. Int. J. Radiat. Biol..

[34] Schaffner F., Ray A.M., Dontenwill M. (2013). Integrin α5β1, the fibronectin receptor, as a pertinent therapeutic target in solid tumors. Cancers.

[35] Blandin A.-F., Renner G., Lehmann M., Lelong-Rebel I., Martin S., Dontenwill M. (2015). β1 integrins as therapeutic targets to disrupt hallmarks of cancer. Front. Pharmacol..

[36] Chen M.B., Lamar J.M., Li R., Hynes R.O., Kamm R.D. (2016). Elucidation of the roles of tumor integrin β1 in the extravasation stage of the metastasis cascade. Cancer Res..

[37] Lahlou H., Muller W.J. (2011). β1-integrins signaling and mammary tumor progression in transgenic mouse models: Implications for human breast cancer. Breast Cancer Res..

[38] Morello V., Cabodi S., Sigismund S., Camacho-Leal M.P., Repetto D., Volante M., Papotti M., Turco E., Defilippi P. (2011). [beta]1 integrin controls EGFR signaling and tumorigenic properties of lung cancer cells. Oncogene.

[39] White D.E., Kurpios N.A., Zuo D., Hassell J.A., Blaess S., Mueller U., Muller W.J. (2004). Targeted disruption of beta1-integrin in a transgenic mouse model of human breast cancer reveals an essential role in mammary tumor induction. Cancer Cell.

[40] Aoudjit F., Vuori K. (2001). Integrin signaling inhibits paclitaxel-induced apoptosis in breast cancer cells. Oncogene.

[41] Hartmann T.N., Burger J.A., Glodek A., Fujii N., Burger M. (2005). CXCR4 chemokine receptor and integrin signaling co-operate in mediating adhesion and chemoresistance in small cell lung cancer (SCLC) cells. Oncogene.

[42] Janouskova H., Maglott A., Leger D.Y., Bossert C., Noulet F., Guerin E., Guenot D., Pinel S., Chastagner P., Plenat F. (2012). Integrin α5β1 plays a critical role in resistance to temozolomide by interfering with the p53 pathway in high-grade glioma. Cancer Res..

[43] Janouskova H., Ray A.-M., Noulet F., Lelong-Rebel I., Choulier L., Schaffner F., Lehmann M., Martin S., Teisinger J., Dontenwill M. (2013). Activation of p53 pathway by Nutlin-3a inhibits the expression of the therapeutic target α5 integrin in colon cancer cells. Cancer Lett..

[44] Klobučar M., Grbčić P., Pavelić S.K., Jonjić N., Visentin S., Sedić M. (2018). Acid ceramidase inhibition sensitizes human colon cancer cells to oxaliplatin through downregulation of transglutaminase 2 and β1 integrin/FAK-mediated signalling. Biochem. Biophys. Res. Commun..

[45] Maglott A., Bartik P., Cosgun S., Klotz P., Rondé P., Fuhrmann G., Takeda K., Martin S., Dontenwill M. (2006). The small alpha5beta1 integrin antagonist, SJ749, reduces proliferation and clonogenicity of human astrocytoma cells. Cancer Res..

[46] Naci D., El Azreq M.-A., Chetoui N., Lauden L., Sigaux F., Charron D., Al-Daccak R., Aoudjit F. (2012). α2β1 integrin promotes chemoresistance against doxorubicin in cancer cells through extracellular signal-regulated kinase (ERK). J. Biol. Chem..

[47] Naci D., Vuori K., Aoudjit F. (2015). Alpha2beta1 integrin in cancer development and chemoresistance. Semin. Cancer Biol..

[48] Pontiggia O., Sampayo R., Raffo D., Motter A., Xu R., Bissell M.J., de Kier Joffé E.B., Simian M. (2012). The tumor microenvironment modulates tamoxifen resistance in breast cancer: A role for soluble stromal factors and fibronectin through β1 integrin. Breast Cancer Res. Treat..

[49] Renner G., Janouskova H., Noulet F., Koenig V., Guerin E., Bär S., Nuesch J., Rechenmacher F., Neubauer S., Kessler H. (2016). Integrin α5β1 and p53 convergent pathways in the control of anti-apoptotic proteins PEA-15 and survivin in high-grade glioma. Cell Death Differ..

[50] Yang D., Shi J., Fu H., Wei Z., Xu J., Hu Z., Zhang Y., Yan R., Cai Q. (2016). Integrinβ1 modulates tumour resistance to gemcitabine and serves as an independent prognostic factor in pancreatic adenocarcinomas. Tumour Biol..

[51] Dickreuter E., Eke I., Krause M., Borgmann K., van Vugt M.A., Cordes N. (2016). Targeting of β1 integrins impairs DNA repair for radiosensitization of head and neck cancer cells. Oncogene.

[52] Eke I., Dickreuter E., Cordes N. (2012). Enhanced radiosensitivity of head and neck squamous cell carcinoma cells by β1 integrin inhibition. Radiother. Oncol..

[53] Eke I., Zscheppang K., Dickreuter E., Hickmann L., Mazzeo E., Unger K., Krause M., Cordes N. (2015). Simultaneous β1 integrin-EGFR targeting and radiosensitization of human head and neck cancer. JNCI J. Natl. Cancer Inst..

[54] Koppenhagen P., Dickreuter E., Cordes N. (2017). Head and neck cancer cell radiosensitization upon dual targeting of c-Abl and beta1-integrin. Radiother. Oncol..

[55] Ahmed K.M., Zhang H., Park C.C. (2013). NF-κB regulates radioresistance mediated by β1-integrin in three-dimensional culture of breast cancer cells. Cancer Res..

[56] Nam J.-M., Onodera Y., Bissell M.J., Park C.C. (2010). Breast cancer cells in three-dimensional culture display an enhanced radioresponse after coordinate targeting of integrin alpha5beta1 and fibronectin. Cancer Res..

[57] Dong X., Luo Z., Liu T., Chai J., Ke Q., Shen L. (2018). Identification of integrin β1 as a novel PAG1-interacting protein involved in the inherent radioresistance of human laryngeal carcinoma. J. Cancer.

[58] Li L., Dong X., Peng F., Shen L. (2018). Integrin β1 regulates the invasion and radioresistance of laryngeal cancer cells by targeting CD147. Cancer Cell Int..

[59] Petrás M., Lajtos T., Friedländer E., Klekner A., Pintye E., Feuerstein B.G., Szöllosi J., Vereb G. (2013). Molecular interactions of ErbB1 (EGFR) and integrin-β1 in astrocytoma frozen sections predict clinical outcome and correlate with Akt-mediated in vitro radioresistance. Neuro Oncol..

[60] Vehlow A., Klapproth E., Storch K., Dickreuter E., Seifert M., Dietrich A., Bütof R., Temme A., Cordes N., Vehlow A. (2017). Adhesion- and stress-related adaptation of glioma radiochemoresistance is circumvented by β1 integrin/JNK co-targeting. Oncotarget.

[61] Moro L., Venturino M., Bozzo C., Silengo L., Altruda F., Beguinot L., Tarone G., Defilippi P. (1998). Integrins induce activation of EGF receptor: Role in MAP kinase induction and adhesion-dependent cell survival. EMBO J..

[62] Miyamoto S., Teramoto H., Gutkind J.S., Yamada K.M. (1996). Integrins can collaborate with growth factors for phosphorylation of receptor tyrosine kinases and MAP kinase activation: Roles of integrin aggregation and occupancy of receptors. J. Cell Biol..

[63] Al-Akhrass H., Naves T., Vincent F., Magnaudeix A., Durand K., Bertin F., Melloni B., Jauberteau M.-O., Lalloué F. (2017). Sortilin limits EGFR signaling by promoting its internalization in lung cancer. Nat. Commun..

[64] Caswell P.T., Chan M., Lindsay A.J., McCaffrey M.W., Boettiger D., Norman J.C. (2008). Rab-coupling protein coordinates recycling of α5β1 integrin and EGFR1 to promote cell migration in 3D microenvironments. J. Cell Biol..

[65] Hang Q., Isaji T., Hou S., Im S., Fukuda T., Gu J. (2015). Integrin α5 suppresses the phosphorylation of epidermal growth factor receptor and its cellular signaling of cell proliferation via N-glycosylation. J. Biol. Chem..

[66] Mattila E., Pellinen T., Nevo J., Vuoriluoto K., Arjonen A., Ivaska J. (2005). Negative regulation of EGFR signalling through integrin-α1β1-mediated activation of protein tyrosine phosphatase TCPTP. Nat. Cell Biol..

[67] Cabodi S., Morello V., Masi A., Cicchi R., Broggio C., Distefano P., Brunelli E., Silengo L., Pavone F., Arcangeli A. (2009). Convergence of integrins and EGF receptor signaling via PI3K/Akt/FoxO pathway in early gene Egr-1 expression. J. Cell. Physiol..

[68] Mocanu M.-M., Fazekas Z., Petrás M., Nagy P., Sebestyén Z., Isola J., Tímár J., Park J.W., Vereb G., Szöllősi J. (2005). Associations of ErbB2, β1-integrin and lipid rafts on Herceptin (Trastuzumab) resistant and sensitive tumor cell lines. Cancer Lett..

[69] Klapproth E., Dickreuter E., Zakrzewski F., Seifert M., Petzold A., Dahl A., Schröck E., Klink B., Cordes N. (2018). Whole exome sequencing identifies mTOR and KEAP1 as potential targets for radiosensitization of HNSCC cells refractory to EGFR and β1 integrin inhibition. Oncotarget.

[70] Zscheppang K., Kurth I., Wachtel N., Dubrovska A., Kunz-Schughart L.A., Cordes N. (2016). Efficacy of Beta1 integrin and EGFR targeting in sphere-forming human head and neck cancer cells. J. Cancer.

[71] Poschau M., Dickreuter E., Singh-Müller J., Zscheppang K., Eke I., Liersch T., Cordes N. (2015). EGFR and β1-integrin targeting differentially affect colorectal carcinoma cell radiosensitivity and invasion. Radiother. Oncol..

[72] Morgillo F., Della Corte C.M., Fasano M., Ciardiello F. (2016). Mechanisms of resistance to EGFR-targeted drugs: Lung cancer. ESMO Open.

[73] Deng Q.-F., SU B., ZHAO Y.-M., TANG L., ZHANG J., ZHOU C.-C. (2016). Integrin β1-mediated acquired gefitinib resistance in non-small cell lung cancer cells occurs via the phosphoinositide 3-kinase-dependent pathway. Oncol. Lett..

[74] Ju L., Zhou C., Li W., Yan L. (2010). Integrin beta1 over-expression associates with resistance to tyrosine kinase inhibitor gefitinib in non-small cell lung cancer. J. Cell. Biochem..

[75] Ju L., Zhou C. (2013). Association of integrin beta1 and c-MET in mediating EGFR TKI gefitinib resistance in non-small cell lung cancer. Cancer Cell Int..

[76] Mousson A., Sick E., Carl P., Dujardin D., De Mey J., Rondé P. (2018). Targeting focal adhesion kinase using inhibitors of protein-protein interactions. Cancers.

[77] Nixon N.A., Hannouf M.B., Verma S. (2018). A review of the value of human epidermal growth factor receptor 2 (HER2)-targeted therapies in breast cancer. Eur. J. Cancer.

[78] Huck L., Pontier S.M., Zuo D.M., Muller W.J. (2010). β1-integrin is dispensable for the induction of ErbB2 mammary tumors but plays a critical role in the metastatic phase of tumor progression. Proc. Natl. Acad. Sci. USA.

[79] Lesniak D., Xu Y., Deschenes J., Lai R., Thoms J., Murray D., Gosh S., Mackey J.R., Sabri S., Abdulkarim B. (2009). Beta1-integrin circumvents the antiproliferative effects of trastuzumab in human epidermal growth factor receptor-2-positive breast cancer. Cancer Res..

[80] Campbell M.R., Zhang H., Ziaee S., Ruiz-Saenz A., Gulizia N., Oeffinger J., Amin D.N., Ahuja D., Moasser M.M., Park C.C. (2016). Effective treatment of HER2-amplified breast cancer by targeting HER3 and β1 integrin. Breast Cancer Res. Treat..

[81] Lewis Phillips G.D., Li G., Dugger D.L., Crocker L.M., Parsons K.L., Mai E., Blättler W.A., Lambert J.M., Chari R.V.J., Lutz R.J. (2008). Targeting HER2-positive breast cancer with trastuzumab-DM1, an antibody-cytotoxic drug conjugate. Cancer Res..

[82] Endo Y., Shen Y., Youssef L.A., Mohan N., Wu W.J. (2018). T-DM1-resistant cells gain high invasive activity via EGFR and integrin cooperated pathways. mAbs.

[83] Sauveur J., Matera E.-L., Chettab K., Valet P., Guitton J., Savina A., Dumontet C. (2018). Esophageal cancer cells resistant to T-DM1 display alterations in cell adhesion and the prostaglandin pathway. Oncotarget.

[84] Mahdi A., Darvishi B., Majidzadeh-A K., Salehi M., Farahmand L. (2019). Challenges facing antiangiogenesis therapy: The significant role of hypoxia-inducible factor and MET in development of resistance to anti-vascular endothelial growth factor-targeted therapies. J. Cell Physiol..

[85] Avraamides C.J., Garmy-Susini B., Varner J.A. (2008). Integrins in angiogenesis and lymphangiogenesis. Nat. Rev. Cancer.

[86] Jahangiri A., Aghi M.K., Carbonell W.S. (2014). β1 Integrin: Critical path to antiangiogenic therapy resistance and beyond. Cancer Res..

[87] Ferrara N., Hillan K.J., Gerber H.-P., Novotny W. (2004). Discovery and development of bevacizumab, an anti-VEGF antibody for treating cancer. Nat. Rev. Drug Discov..

[88] DeLay B.M., Jahangiri A., Carbonell W.S., Hu Y.-L., Tsao S., Tom M.W., Paquette J., Tokuyasu T.A., Aghi M.K. (2012). Microarray analysis verifies two distinct phenotypes of glioblastomas resistant to anti-angiogenic therapy. Clin. Cancer Res..

[89] Shojaei F., Lee J.H., Simmons B.H., Wong A., Esparza C.O., Plumlee P.A., Feng J., Stewart A.E., Hu-Lowe D.D., Christensen J.G. (2010). HGF/c-Met acts as an alternative angiogenic pathway in sunitinib-resistant tumors. Cancer Res..

[90] Mitra A.K., Sawada K., Tiwari P., Mui K., Gwin K., Lengyel E. (2011). Ligand-independent activation of c-Met by fibronectin and α(5)β(1)-integrin regulates ovarian cancer invasion and metastasis. Oncogene.

[91] Hongu T., Yamauchi Y., Funakoshi Y., Katagiri N., Ohbayashi N., Kanaho Y. (2016). Pathological functions of the small GTPase Arf6 in cancer progression: Tumor angiogenesis and metastasis. Small GTPases.

[92] Mai A., Muharram G., Barrow-McGee R., Baghirov H., Rantala J., Kermorgant S., Ivaska J. (2014). Distinct c-Met activation mechanisms induce cell rounding or invasion through pathways involving integrins, RhoA and HIP1. J. Cell Sci..

[93] Alanko J., Mai A., Jacquemet G., Schauer K., Kaukonen R., Saari M., Goud B., Ivaska J. (2015). Integrin endosomal signalling suppresses anoikis. Nat. Cell Biol..

[94] Tripolitsioti D., Kumar K.S., Neve A., Migliavacca J., Capdeville C., Rushing E.J., Ma M., Kijima N., Sharma A., Pruschy M. (2018). MAP4K4 controlled integrin β1 activation and c-Met endocytosis are associated with invasive behavior of medulloblastoma cells. Oncotarget.

[95] Barrow-McGee R., Kishi N., Joffre C., Ménard L., Hervieu A., Bakhouche B.A., Noval A.J., Mai A., Guzmán C., Robbez-Masson L. (2016). Beta 1-integrin-c-Met cooperation reveals an inside-in survival signalling on autophagy-related endomembranes. Nat. Commun..

[96] Weis S.M., Cheresh D.A. (2011). αv integrins in angiogenesis and cancer. Cold Spring Harb. Perspect. Med..

[97] Nieberler M., Reuning U., Reichart F., Notni J., Wester H.-J., Schwaiger M., Weinmüller M., Räder A., Steiger K., Kessler H. (2017). Exploring the role of RGD-recognizing integrins in cancer. Cancers.

[98] He J., Wang F., Qi H., Li Y., Liang H. (2009). Down-regulation of αv integrin by retroviral delivery of small interfering RNA reduces multicellular resistance of HT29. Cancer Lett..

[99] Maubant S., Cruet-Hennequart S., Poulain L., Carreiras F., Sichel F., Luis J., Staedel C., Gauduchon P. (2002). Altered adhesion properties and alpha v integrin expression in a cisplatin-resistant human ovarian carcinoma cell line. Int. J. Cancer.

[100] Malric L., Monferran S., Delmas C., Arnauduc F., Dahan P., Boyrie S., Deshors P., Lubrano V., Da Mota D.F., Gilhodes J. (2019). Inhibiting integrin β8 to differentiate and radiosensitize glioblastoma-initiating cells. Mol. Cancer Res..

[101] Mikkelsen T., Brodie C., Finniss S., Berens M.E., Rennert J.L., Nelson K., Lemke N., Brown S.L., Hahn D., Neuteboom B. (2009). Radiation sensitization of glioblastoma by cilengitide has unanticipated schedule-dependency. Int. J. Cancer.

[102] Monferran S., Skuli N., Delmas C., Favre G., Bonnet J., Cohen-Jonathan-Moyal E., Toulas C. (2008). Alphavbeta3 and alphavbeta5 integrins control glioma cell response to ionising radiation through ILK and RhoB. Int. J. Cancer.

[103] Ning S., Tian J., Marshall D.J., Knox S.J. (2010). Anti–αv integrin monoclonal antibody intetumumab enhances the efficacy of radiation therapy and reduces metastasis of human cancer xenografts in nude rats. Cancer Res..

[104] Ou J., Luan W., Deng J., Sa R., Liang H. (2012). αV integrin induces multicellular radioresistance in human nasopharyngeal carcinoma via activating SAPK/JNK pathway. PLoS ONE.

[105] Cai W., Chen X. (2006). Anti-angiogenic cancer therapy based on integrin alphavbeta3 antagonism. Anticancer Agents Med. Chem..

[106] Hsu A.R., Veeravagu A., Cai W., Hou L.C., Tse V., Chen X. (2007). Integrin alpha v beta 3 antagonists for anti-angiogenic cancer treatment. Recent Pat. Anticancer Drug Discov..

[107] Zhang D., Pier T., McNeel D.G., Wilding G., Friedl A. (2007). Effects of a monoclonal anti-alphavbeta3 integrin antibody on blood vessels - a pharmacodynamic study. Invest. New Drugs.

[108] Eskens F.A.L.M., Dumez H., Hoekstra R., Perschl A., Brindley C., Böttcher S., Wynendaele W., Drevs J., Verweij J., van Oosterom A.T. (2003). Phase I and pharmacokinetic study of continuous twice weekly intravenous administration of Cilengitide (EMD 121974), a novel inhibitor of the integrins alphavbeta3 and alphavbeta5 in patients with advanced solid tumours. Eur. J. Cancer.

[109] Mas-Moruno C., Rechenmacher F., Kessler H. (2010). Cilengitide: The first anti-angiogenic small molecule drug candidate. design, synthesis and clinical evaluation. Anticancer Agents Med. Chem..

[110] Stupp R., Hegi M.E., Neyns B., Goldbrunner R., Schlegel U., Clement P.M.J., Grabenbauer G.G., Ochsenbein A.F., Simon M., Dietrich P.-Y. (2010). Phase I/IIa study of cilengitide and temozolomide with concomitant radiotherapy followed by cilengitide and temozolomide maintenance therapy in patients with newly diagnosed glioblastoma. J. Clin. Oncol..

[111] Khasraw M., Lee A., McCowatt S., Kerestes Z., Buyse M.E., Back M., Kichenadasse G., Ackland S., Wheeler H. (2016). Cilengitide with metronomic temozolomide, procarbazine, and standard radiotherapy in patients with glioblastoma and unmethylated MGMT gene promoter in ExCentric, an open-label phase II trial. J. Neurooncol..

[112] Nabors L.B., Fink K.L., Mikkelsen T., Grujicic D., Tarnawski R., Nam D.H., Mazurkiewicz M., Salacz M., Ashby L., Zagonel V. (2015). Two cilengitide regimens in combination with standard treatment for patients with newly diagnosed glioblastoma and unmethylated MGMT gene promoter: Results of the open-label, controlled, randomized phase II CORE study. Neuro Oncol..

[113] Stupp R., Hegi M.E., Gorlia T., Erridge S.C., Perry J., Hong Y.-K., Aldape K.D., Lhermitte B., Pietsch T., Grujicic D. (2014). Cilengitide combined with standard treatment for patients with newly diagnosed glioblastoma with methylated MGMT promoter (CENTRIC EORTC 26071-22072 study): A multicentre, randomised, open-label, phase 3 trial. Lancet Oncol..

[114] Cosset É., Ilmjärv S., Dutoit V., Elliott K., von Schalscha T., Camargo M.F., Reiss A., Moroishi T., Seguin L., Gomez G. (2017). Glut3 addiction is a druggable vulnerability for a molecularly defined subpopulation of glioblastoma. Cancer Cell.

[115] Desgrosellier J.S., Barnes L.A., Shields D.J., Huang M., Lau S.K., Prévost N., Tarin D., Shattil S.J., Cheresh D.A. (2009). Integrin αvβ3/c-src “Oncogenic Unit” promotes anchorage-independence and tumor progression. Nat. Med..

[116] Seguin L., Camargo M.F., Wettersten H.I., Kato S., Desgrosellier J.S., von Schalscha T., Elliott K.C., Cosset E., Lesperance J., Weis S.M. (2017). Galectin-3, a druggable vulnerability for KRAS-addicted cancers. Cancer Discov..

[117] Wdowiak K., Francuz T., Gallego-Colon E., Ruiz-Agamez N., Kubeczko M., Grochoła I., Wojnar J. (2018). Galectin targeted therapy in oncology: Current knowledge and perspectives. Int. J. Mol. Sci..

[118] Chen X., Zhu L., Ma Z., Sun G., Luo X., Li M., Zhai S., Li P., Wang X. (2015). Oncogenic miR-9 is a target of erlotinib in NSCLCs. Sci. Rep..

[119] Gao Y., Fan X., Li W., Ping W., Deng Y., Fu X. (2014). miR-138-5p reverses gefitinib resistance in non-small cell lung cancer cells via negatively regulating G protein-coupled receptor 124. Biochem. Biophys. Res. Commun..

[120] Li B., Ren S., Li X., Wang Y., Garfield D., Zhou S., Chen X., Su C., Chen M., Kuang P. (2014). MiR-21 overexpression is associated with acquired resistance of EGFR-TKI in non-small cell lung cancer. Lung Cancer.

[121] Shen H., Zhu F., Liu J., Xu T., Pei D., Wang R., Qian Y., Li Q., Wang L., Shi Z. (2014). Alteration in Mir-21/PTEN Expression Modulates Gefitinib Resistance in Non-Small Cell Lung Cancer. PLoS ONE.

[122] Wang S., Su X., Bai H., Zhao J., Duan J., An T., Zhuo M., Wang Z., Wu M., Li Z. (2015). Identification of plasma microRNA profiles for primary resistance to EGFR-TKIs in advanced non-small cell lung cancer (NSCLC) patients with EGFR activating mutation. J. Hematol. Oncol..

[123] Yan G., Yao R., Tang D., Qiu T., Shen Y., Jiao W., Ge N., Xuan Y., Wang Y. (2014). Prognostic significance of microRNA expression in completely resected lung adenocarcinoma and the associated response to erlotinib. Med. Oncol..

[124] Zhang N., Li Y., Zheng Y., Zhang L., Pan Y., Yu J., Yang M. (2019). miR-608 and miR-4513 significantly contribute to the prognosis of lung adenocarcinoma treated with EGFR-TKIs. Lab. Invest..

[125] Vansteenkiste J., Barlesi F., Waller C.F., Bennouna J., Gridelli C., Goekkurt E., Verhoeven D., Szczesna A., Feurer M., Milanowski J. (2015). Cilengitide combined with cetuximab and platinum-based chemotherapy as first-line treatment in advanced non-small-cell lung cancer (NSCLC) patients: Results of an open-label, randomized, controlled phase II study (CERTO). Ann. Oncol..

[126] Cedra S., Wiegand S., Kolb M., Dietz A., Wichmann G. (2017). Reduced cytokine release in ex vivo response to cilengitide and cetuximab is a marker for improved survival of head and neck cancer patients. Cancers (Basel).

[127] Wichmann G., Cedra S., Schlegel D., Kolb M., Wiegand S., Boehm A., Hofer M., Dietz A. (2017). cilengitide and cetuximab reduce cytokine production and colony formation of head and neck squamous cell carcinoma cells ex vivo. Anticancer Res..

[128] Élez E., Kocáková I., Höhler T., Martens U.M., Bokemeyer C., Van Cutsem E., Melichar B., Smakal M., Csőszi T., Topuzov E. (2015). Abituzumab combined with cetuximab plus irinotecan versus cetuximab plus irinotecan alone for patients with KRAS wild-type metastatic colorectal cancer: The randomised phase I/II POSEIDON trial. Ann. Oncol..

[129] Maki R.G. (2010). Small is beautiful: insulin-like growth factors and their role in growth, development, and cancer. J. Clin. Oncol..

[130] Chen H.X., Sharon E. (2013). IGF-1R as an anti-cancer target—trials and tribulations. Chin. J. Cancer.

[131] Saegusa J., Yamaji S., Ieguchi K., Wu C.-Y., Lam K.S., Liu F.-T., Takada Y.K., Takada Y. (2009). The direct binding of insulin-like growth factor-1 (igf-1) to integrin αvβ3 is involved in igf-1 signaling. J. Biol. Chem..

[132] Fujita M., Takada Y.K., Takada Y. (2013). Insulin-like growth factor (IGF) signaling requires αvβ3-IGF1-IGF type 1 receptor (IGF1R) ternary complex formation in anchorage independence, and the complex formation does not require IGF1R and Src activation. J. Biol. Chem..

[133] Fujita M., Ieguchi K., Cedano-Prieto D.M., Fong A., Wilkerson C., Chen J.Q., Wu M., Lo S.-H., Cheung A.T.W., Wilson M.D. (2013). An integrin binding-defective mutant of insulin-like growth factor-1 (R36E/R37E IGF1) acts as a dominant-negative antagonist of the IGF1 receptor (IGF1R) and suppresses tumorigenesis but still binds to IGF1R. J. Biol. Chem..

[134] Takada Y., Takada Y.K., Fujita M. (2017). Crosstalk between insulin-like growth factor (IGF) receptor and integrins through direct integrin binding to IGF1. Cytokine Growth Factor Rev..

[135] Hasskarl J. (2010). Sorafenib. Recent Results Cancer Res..

[136] Chong Y., Tang D., Xiong Q., Jiang X., Xu C., Huang Y., Wang J., Zhou H., Shi Y., Wu X. (2016). Galectin-1 from cancer-associated fibroblasts induces epithelial-mesenchymal transition through β1 integrin-mediated upregulation of Gli1 in gastric cancer. J. Exp. Clin. Cancer Res..

[137] Nam K., Son S., Oh S., Jeon D., Kim H., Noh D.-Y., Kim S., Shin I. (2017). Binding of galectin-1 to integrin β1 potentiates drug resistance by promoting survivin expression in breast cancer cells. Oncotarget.

[138] He X.-J., Tao H.-Q., Hu Z.-M., Ma Y.-Y., Xu J., Wang H.-J., Xia Y.-J., Li L., Fei B.-Y., Li Y.-Q. (2014). Expression of galectin-1 in carcinoma-associated fibroblasts promotes gastric cancer cell invasion through upregulation of integrin β1. Cancer Sci..

[139] Liebert M., Washington R., Wedemeyer G., Carey T.E., Grossman H.B. (1994). Loss of co-localization of alpha 6 beta 4 integrin and collagen VII in bladder cancer. Am. J. Pathol..

[140] Rodius S., Indra G., Thibault C., Pfister V., Georges-Labouesse E. (2007). Loss of alpha6 integrins in keratinocytes leads to an increase in TGFbeta and AP1 signaling and in expression of differentiation genes. J. Cell. Physiol..

[141] Raymond K., Kreft M., Janssen H., Calafat J., Sonnenberg A. (2005). Keratinocytes display normal proliferation, survival and differentiation in conditional β4-integrin knockout mice. J. Cell. Sci..

[142] Faure E., Garrouste F., Parat F., Monferran S., Leloup L., Pommier G., Kovacic H., Lehmann M. (2012). P2Y2 receptor inhibits EGF-induced MAPK pathway to stabilise keratinocyte hemidesmosomes. J. Cell. Sci..

[143] Frijns E., Sachs N., Kreft M., Wilhelmsen K., Sonnenberg A. (2010). EGF-induced MAPK signaling inhibits hemidesmosome formation through phosphorylation of the integrin {beta}4. J. Biol. Chem.

[144] Frijns E., Kuikman I., Litjens S., Raspe M., Jalink K., Ports M., Wilhelmsen K., Sonnenberg A. (2012). Phosphorylation of threonine 1736 in the C-terminal tail of integrin β4 contributes to hemidesmosome disassembly. Mol. Biol. Cell.

[145] Margadant C., Frijns E., Wilhelmsen K., Sonnenberg A. (2008). Regulation of hemidesmosome disassembly by growth factor receptors. Curr. Opin. Cell Biol..

[146] Mariotti A., Kedeshian P.A., Dans M., Curatola A.M., Gagnoux-Palacios L., Giancotti F.G. (2001). EGF-R signaling through Fyn kinase disrupts the function of integrin alpha6beta4 at hemidesmosomes: Role in epithelial cell migration and carcinoma invasion. J. Cell Biol..

[147] Wilhelmsen K., Litjens S.H.M., Kuikman I., Margadant C., van Rheenen J., Sonnenberg A. (2007). Serine phosphorylation of the integrin β4 subunit is necessary for epidermal growth factor receptor–induced hemidesmosome disruption. Mol. Biol. Cell.

[148] Ramovs V., Te Molder L., Sonnenberg A. (2017). The opposing roles of laminin-binding integrins in cancer. Matrix Biol..

[149] Stewart R.L., O’Connor K.L. (2015). Clinical significance of the integrin α6β4 in human malignancies. Lab. Invest..

[150] De Arcangelis A., Hamade H., Alpy F., Normand S., Bruyère E., Lefebvre O., Méchine-Neuville A., Siebert S., Pfister V., Lepage P. (2017). Hemidesmosome integrity protects the colon against colitis and colorectal cancer. Gut.

[151] Laval S., Laklai H., Fanjul M., Pucelle M., Laurell H., Billon-Galés A., Le Guellec S., Delisle M.-B., Sonnenberg A., Susini C. (2014). Dual roles of hemidesmosomal proteins in the pancreatic epithelium: The phosphoinositide 3-kinase decides. Oncogene.

[152] Yu P.T., Babicky M., Jaquish D., French R., Marayuma K., Mose E., Niessen S., Hoover H., Shields D., Cheresh D. (2012). The RON-receptor regulates pancreatic cancer cell migration through phosphorylation-dependent breakdown of the hemidesmosome. Int. J. Cancer.

[153] Trusolino L., Bertotti A., Comoglio P.M. (2001). A Signaling Adapter Function for α6β4 Integrin in the Control of HGF-Dependent Invasive Growth. Cell.

[154] Scartozzi M., Giampieri R., Loretelli C., Mandolesi A., del Prete M., Biagetti S., Alfonsi S., Faloppi L., Bianconi M., Bittoni A. (2013). Role of β4 integrin in HER-3-negative, K-RAS wild-type metastatic colorectal tumors receiving cetuximab. Future Oncol..

[155] Martins Cavaco A.C., Rezaei M., Caliandro M.F., Martins Lima A., Stehling M., Dhayat S.A., Haier J., Brakebusch C., Eble J.A. (2018). The interaction between laminin-332 and α3β1 integrin determines differentiation and maintenance of CAFs, and supports invasion of pancreatic duct adenocarcinoma cells. Cancers (Basel).

[156] Kalluri R. (2016). The biology and function of fibroblasts in cancer. Nat. Rev. Cancer.

[157] Fiori M.E., Di Franco S., Villanova L., Bianca P., Stassi G., De Maria R. (2019). Cancer-associated fibroblasts as abettors of tumor progression at the crossroads of EMT and therapy resistance. Mol. Cancer.

[158] Daverey A., Drain A.P., Kidambi S. (2015). Physical intimacy of breast cancer cells with mesenchymal stem cells elicits trastuzumab resistance through Src activation. Sci. Rep..

[159] Marusyk A., Tabassum D.P., Janiszewska M., Place A.E., Trinh A., Rozhok A.I., Pyne S., Guerriero J.L., Shu S., Ekram M. (2016). Spatial proximity to fibroblasts impacts molecular features and therapeutic sensitivity of breast cancer cells influencing clinical outcomes. Cancer Res..

[160] McFarlane S., McFarlane C., Montgomery N., Hill A., Waugh D.J.J. (2015). CD44-mediated activation of α5β1-integrin, cortactin and paxillin signaling underpins adhesion of basal-like breast cancer cells to endothelium and Fibronectin-enriched matrices. Oncotarget.

[161] Blandin A.-F., Noulet F., Renner G., Mercier M.-C., Choulier L., Vauchelles R., Ronde P., Carreiras F., Etienne-Selloum N., Vereb G. (2016). Glioma cell dispersion is driven by α5 integrin-mediated cell–matrix and cell–cell interactions. Cancer Lett..

[162] Gao Q., Yang Z., Xu S., Li X., Yang X., Jin P., Liu Y., Zhou X., Zhang T., Gong C. (2019). Heterotypic CAF-tumor spheroids promote early peritoneal metastatis of ovarian cancer. J. Exp. Med..

[163] Wang Y., Zhang T., Guo L., Ren T., Yang Y. (2019). Stromal extracellular matrix is a microenvironmental cue promoting resistance to EGFR tyrosine kinase inhibitors in lung cancer cells. Int. J. Biochem. Cell Biol..

[164] Yamazaki S., Higuchi Y., Ishibashi M., Hashimoto H., Yasunaga M., Matsumura Y., Tsuchihara K., Tsuboi M., Goto K., Ochiai A. (2018). Collagen type I induces EGFR-TKI resistance in EGFR-mutated cancer cells by mTOR activation through Akt-independent pathway. Cancer Sci..

[165] Brighton H.E., Angus S.P., Bo T., Roques J., Tagliatela A.C., Darr D.B., Karagoz K., Sciaky N., Gatza M.L., Sharpless N.E. (2018). New mechanisms of resistance to MEK inhibitors in melanoma revealed by intravital imaging. Cancer Res..

[166] Hirata E., Girotti M.R., Viros A., Hooper S., Spencer-Dene B., Matsuda M., Larkin J., Marais R., Sahai E. (2015). Intravital imaging reveals how BRAF inhibition generates drug-tolerant microenvironments with high integrin β1/FAK signaling. Cancer Cell.

[167] Fedorenko I.V., Abel E.V., Koomen J.M., Fang B., Wood E.R., Chen Y.A., Fisher K.J., Iyengar S., Dahlman K.B., Wargo J.A. (2016). Fibronectin induction abrogates the BRAF inhibitor response of BRAF V600E/PTEN-null melanoma cells. Oncogene.

[168] Margue C., Philippidou D., Kozar I., Cesi G., Felten P., Kulms D., Letellier E., Haan C., Kreis S. (2019). Kinase inhibitor library screening identifies synergistic drug combinations effective in sensitive and resistant melanoma cells. J. Exp. Clin. Cancer Res..

[169] Dupont S. (2016). Role of YAP/TAZ in cell-matrix adhesion-mediated signalling and mechanotransduction. Exp. Cell Res..

[170] Elbediwy A., Thompson B.J. (2018). Evolution of mechanotransduction via YAP/TAZ in animal epithelia. Current Opin. Cell Biol..

[171] Kapoor A., Yao W., Ying H., Hua S., Liewen A., Wang Q., Zhong Y., Wu C.-J., Sadanandam A., Hu B. (2014). Yap1 activation enables bypass of oncogenic Kras addiction in pancreatic cancer. Cell.

[172] Shao D.D., Xue W., Krall E.B., Bhutkar A., Piccioni F., Wang X., Schinzel A.C., Sood S., Rosenbluh J., Kim J.W. (2014). KRAS and YAP1 converge to regulate EMT and tumor survival. Cell.

[173] Kim M.H., Kim J., Hong H., Lee S.-H., Lee J.-K., Jung E., Kim J. (2016). Actin remodeling confers BRAF inhibitor resistance to melanoma cells through YAP/TAZ activation. EMBO J..

[174] Lin L., Sabnis A.J., Chan E., Olivas V., Cade L., Pazarentzos E., Asthana S., Neel D., Yan J.J., Lu X. (2015). The Hippo effector YAP promotes resistance to RAF- and MEK-targeted cancer therapies. Nat. Genet..

[175] Chang C.-C., Hsieh T.-L., Tiong T.-Y., Hsiao C.-H., Ji A.T.-Q., Hsu W.-T., Lee O.K., Ho J.H. (2015). Regulation of metastatic ability and drug resistance in pulmonary adenocarcinoma by matrix rigidity via activating c-Met and EGFR. Biomaterials.

[176] Lin C.-H., Pelissier F.A., Zhang H., Lakins J., Weaver V.M., Park C., LaBarge M.A. (2015). Microenvironment rigidity modulates responses to the HER2 receptor tyrosine kinase inhibitor lapatinib via YAP and TAZ transcription factors. Mol. Biol. Cell.

[177] Nguyen T.V., Sleiman M., Moriarty T., Herrick W.G., Peyton S.R. (2014). Sorafenib resistance and JNK signaling in carcinoma during extracellular matrix stiffening. Biomaterials.

[178] Attieh Y., Clark A.G., Grass C., Richon S., Pocard M., Mariani P., Elkhatib N., Betz T., Gurchenkov B., Vignjevic D.M. (2017). Cancer-associated fibroblasts lead tumor invasion through integrin-β3-dependent fibronectin assembly. J. Cell Biol..

[179] Erdogan B., Ao M., White L.M., Means A.L., Brewer B.M., Yang L., Washington M.K., Shi C., Franco O.E., Weaver A.M. (2017). Cancer-associated fibroblasts promote directional cancer cell migration by aligning fibronectin. J. Cell Biol..

[180] Gaggioli C., Hooper S., Hidalgo-Carcedo C., Grosse R., Marshall J.F., Harrington K., Sahai E. (2007). Fibroblast-led collective invasion of carcinoma cells with differing roles for RhoGTPases in leading and following cells. Nat. Cell Biol..

[181] Goetz J.G., Minguet S., Navarro-Lérida I., Lazcano J.J., Samaniego R., Calvo E., Tello M., Osteso-Ibáñez T., Pellinen T., Echarri A. (2011). Biomechanical remodeling of the microenvironment by stromal caveolin-1 favors tumor invasion and metastasis. Cell.

[182] Navab R., Strumpf D., To C., Pasko E., Kim K.S., Park C.J., Hai J., Liu J., Jonkman J., Barczyk M. (2016). Integrin α11β1 regulates cancer stromal stiffness and promotes tumorigenicity and metastasis in non-small cell lung cancer. Oncogene.

[183] Levental K.R., Yu H., Kass L., Lakins J.N., Egeblad M., Erler J.T., Fong S.F.T., Csiszar K., Giaccia A., Weninger W. (2009). Matrix crosslinking forces tumor progression by enhancing integrin signaling. Cell.

[184] Alkasalias T., Moyano-Galceran L., Arsenian-Henriksson M., Lehti K. (2018). Fibroblasts in the tumor microenvironment: Shield or spear?. Int. J. Mol. Sci..

[185] Cooper J., Giancotti F.G. (2019). Integrin signaling in Cancer: Mechanotransduction, stemness, epithelial plasticity, and therapeutic resistance. Cancer Cell.

[186] Löffek S., Franzke C.-W., Helfrich I. (2016). Tension in cancer. Int. J. Mol. Sci..

[187] Mercier M.-C., Dontenwill M., Choulier L. (2017). Selection of nucleic acid aptamers targeting tumor cell-surface protein biomarkers. Cancers (Basel).

[188] Camorani S., Crescenzi E., Gramanzini M., Fedele M., Zannetti A., Cerchia L. (2017). Aptamer-mediated impairment of EGFR-integrin αvβ3 complex inhibits vasculogenic mimicry and growth of triple-negative breast cancers. Sci. Rep..

[189] Laurenzana A., Margheri F., Biagioni A., Chillà A., Pimpinelli N., Ruzzolini J., Peppicelli S., Andreucci E., Calorini L., Serratì S. (2019). EGFR/uPAR interaction as druggable target to overcome vemurafenib acquired resistance in melanoma cells. EBioMedicine.

